# Reactomes of Porcine Alveolar Macrophages Infected with Porcine Reproductive and Respiratory Syndrome Virus

**DOI:** 10.1371/journal.pone.0059229

**Published:** 2013-03-19

**Authors:** Zhihua Jiang, Xiang Zhou, Jennifer J. Michal, Xiao-Lin Wu, Lifan Zhang, Ming Zhang, Bo Ding, Bang Liu, Valipuram S. Manoranjan, John D. Neill, Gregory P. Harhay, Marcus E. Kehrli, Laura C. Miller

**Affiliations:** 1 Department of Animal Sciences, Washington State University, Pullman, Washington, United States of America; 2 Department of Dairy Science, University of Wisconsin-Madison, Madison, Wisconsin, United States of America; 3 College of Animal Science and Technology, Huazhong Agricultural University, Hubei, China; 4 Department of Mathematics, Washington State University, Pullman, Washington, United States of America; 5 Ruminant Diseases and Immunology Research Unit, United States Department of Agriculture, Agricultural Research Service, National Animal Disease Center, Ames, Iowa, United States of America; 6 Animal Health Research Unit, United States Meat Animal Research Center, United States Department of Agriculture, Agricultural Research Service, Clay Center, Nebraska, United States of America; 7 Virus and Prion Research Unit, National Animal Disease Center, United States Department of Agriculture, Agricultural Research Service, Ames, Iowa, United States of America; Virginia Polytechnic Institute and State University, United States of America

## Abstract

Porcine reproductive and respiratory syndrome (PRRS) has devastated pig industries worldwide for many years. It is caused by a small RNA virus (PRRSV), which targets almost exclusively pig monocytes or macrophages. In the present study, five SAGE (serial analysis of gene expression) libraries derived from 0 hour mock-infected and 6, 12, 16 and 24 hours PRRSV-infected porcine alveolar macrophages (PAMs) produced a total 643,255 sequenced tags with 91,807 unique tags. Differentially expressed (DE) tags were then detected using the Bayesian framework followed by gene/mRNA assignment, arbitrary selection and manual annotation, which determined 699 DE genes for reactome analysis. The DAVID, KEGG and REACTOME databases assigned 573 of the DE genes into six biological systems, 60 functional categories and 504 pathways. The six systems are: cellular processes, genetic information processing, environmental information processing, metabolism, organismal systems and human diseases as defined by KEGG with modification. Self-organizing map (SOM) analysis further grouped these 699 DE genes into ten clusters, reflecting their expression trends along these five time points. Based on the number one functional category in each system, cell growth and death, transcription processes, signal transductions, energy metabolism, immune system and infectious diseases formed the major reactomes of PAMs responding to PRRSV infection. Our investigation also focused on dominant pathways that had at least 20 DE genes identified, multi-pathway genes that were involved in 10 or more pathways and exclusively-expressed genes that were included in one system. Overall, our present study reported a large set of DE genes, compiled a comprehensive coverage of pathways, and revealed system-based reactomes of PAMs infected with PRRSV. We believe that our reactome data provides new insight into molecular mechanisms involved in host genetic complexity of antiviral activities against PRRSV and lays a strong foundation for vaccine development to control PRRS incidence in pigs.

## Introduction

Porcine reproductive and respiratory syndrome (PRRS), also known as Mystery Swine Disease, Blue Ear Disease, Porcine Endemic Abortion and Respiratory Syndrome (PEARS) and Swine Infertility Respiratory Syndrome (SIRS), was first reported in USA in 1987 and in Europe in 1990 [1], [2]. Since then, PRRS has devastated the pig industries of many countries and has become the most economically important disease in pigs worldwide. A recent study estimated that PRRS costs the pork industry $664 million per year in the United States of America (http://www.pork.org/News).

The disease is caused by a small RNA virus (PRRSV) classified in the order Nidovirales, family Arteriviridae, and genus Arterivirus. PRRSV causes severe reproductive failure of the sow, including third-trimester abortions, early farrowing with stillborns, mummies, neonatal death and weak piglets, agalactia and mastitis, and prolonged anoestrus and delayed return to estrus post-weaning. Respiratory disease is the major clinical sign in neonatal pigs and is characterized by fever, interstitial pneumonia, eyelid edema, periocular edema, blue discoloration of the ears and shaking [3], [4]. The mortality in neonatal pigs infected with PRRSV can reach 100%. In growing/finishing pigs, subclinical infection is much more common. Some PRRSV-infected boars demonstrate a loss of libido, lethargy, lowered sperm volume and decreased fertility.

PRRSV has remarkable genetic variation with two distinct genetic and antigenic groups: Type 1 (European) and Type 2 (North American), which only share 60% nucleotide identity [5]. In 2006, previously unparalleled large-scale outbreaks of highly-pathogenic PRRS, also named “Blue Ear” or “high fever” disease, occurred in China. It spread to more than 10 provinces (autonomous cities or regions) and affected over 2 million pigs with about 400,000 fatal cases [6]. Best estimates suggest that at least 50 million pigs were affected [7]. Since then, highly-pathogenic PRRS outbreaks were also reported in 2007 and 2008 in other Asian countries, such as Vietnam and the Philippines [8]. These data clearly indicate that PRRSV is able to mutate, thus causing challenges in effective vaccine development. For example, while modified live-attenuated vaccines and inactivated vaccines against PRRSV have been available for many years, none of them can prevent respiratory infection, transmission, or pig-to-pig transmission of virus. In particular, modified-live vaccines are generally effective against homologous strains but variable in success against heterologous strains, while efficacy of inactivated vaccines in the field is more limited and restricted to homologous strains [9]. In addition, PRRSV has developed diverse mechanisms to evade porcine antiviral immune responses [10]. Once the virus infects pig tissues, it has several mechanisms to evade the pig’s immune system, causing a several week delay in protective antibody production [11]–[13]. In the absence of control efforts, the virus will persist indefinitely in swine herds.

PRRSV targets almost exclusively pig monocytes or macrophages [14], [15]. The entry of PRRSV into porcine alveolar macrophages (PAMs) is proposed to include four steps [16]. First, the PRRSV virion attaches to heparan sulphate glycosaminoglycans on the macrophage surface. Second, the virus then forms a more stable binding with the sialoadhesin receptor via sialic acid residues associated with M/GP_5_ glycoprotein complexes present in the viral envelope. Third, following attachment to sialoadhesin, the virus–receptor complex is endocytosed via clathrin-coated vesicles. Once endocytosed, viral genome release is dependent on endosomal acidification. There appears to be involvement of CD163 with viral genome release that is possible through interactions with the viral glycoproteins, GP2 and GP4 and that is dependent upon a function CD-163 scavenger receptor cysteine rich domain 5 being present. In addition, several proteases have been implicated in this final step of PRRSV entry into macrophages. Once the genome is released into the cytoplasm of the host cell, virus transcriptional and translational events required for the formation of new virions are initiated. Here we report the reactome dynamics of PAMs in response to PRRSV infection *in vitro,* following serial analysis of gene expression (SAGE) [17], in order to reveal the host transcriptional events in response to virus replication and cellular resistance, thus providing new insights into molecular mechanisms involved in the cellular complexity of antiviral activities against PRRSV.

## Results

### Reactome of PAMs Infected with PRRSV: Snapshots

**Figure 1:**
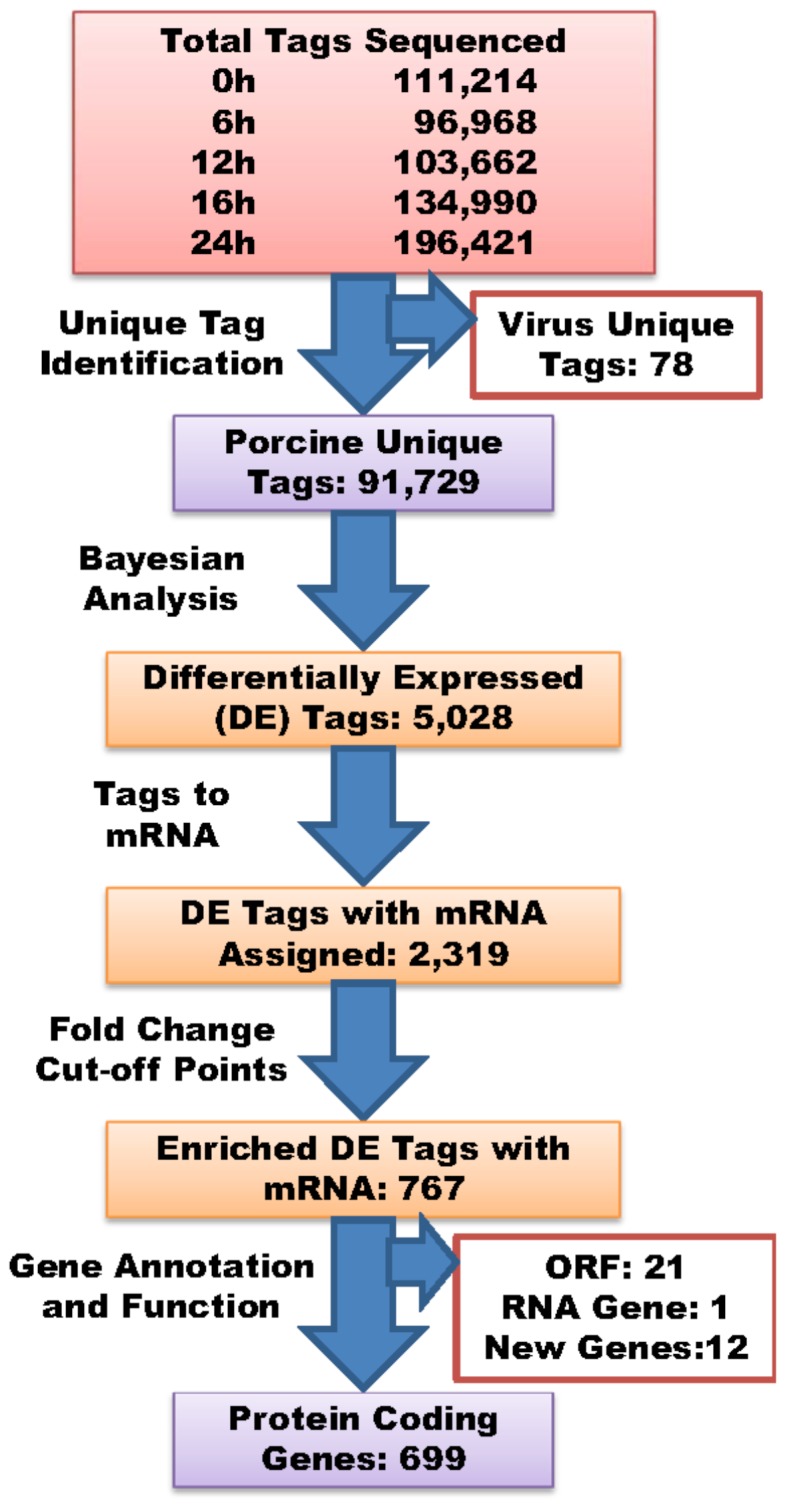
Identification and characterization of tags/genes differentially expressed between the 0 hour mock-infected and the 6, 12, 16 and 24 hours PRRSV-infected PAM cells.

In SAGE analysis, a set of “tag” fragments (13–15 bp in size) derived from restriction positions of cDNA molecules are pooled, collected, sequenced and assigned to genes/transcripts. Five SAGE libraries constructed from the 0 hour mock-infected and 6, 12, 16 and 24 hour PRRSV-infected cells produced a total of 643,255 sequenced tags, which allowed identification of 91,807 unique tags among these five time points (Figure 1). As PAMs were infected with PRRSV, we anticipated the existence of viral mRNA tags in the cells. In fact, the virus complete genome sequence contains a total of 74 cut sites for restriction enzyme *Nla*III. Using the complete genome sequence of PRRSV strain SD1-100 (GQ914997.1) as a reference, we discovered a total of 78 tentative virus tags, including 46 derived from the sense strand and 32 from the antisense strand (Table S1). The total count for all of these virus-specific tags was 0 in the 0 hour mock-infected cells, but reached 267, 11,270, 7,854 and 3,770 copies in the 6, 12, 16 and 24 hour PRRSV-infected cell libraries, respectively. The most abundantly expressed tag was the 3′-most cut site (CGGCCGAAAT) (Table S1), having 225 (84.27% of 267), 9,500 (84.29% of 11270), 6,902 (87.88% of 7,854) and 3,622 (96.07% of 3,770) copies sequenced in PAMs infected with PRRSV for 6, 12, 16 and 24 hours. Virus tags accounted for 9.16% of total tags (9,500/103,662 tags) at 12 hours post infection; therefore, we deleted all virus tags from each library and re-calculated the number of tags per million (TPM) for each host gene tag.

Compared to the 0 hour mock-infected cells, Bayesian analysis revealed that PRRSV-infected cells had 891, 972, 1,230 and 1,323 down- and 1,201, 1,199, 1,276 and 1,042 up-regulated DE tags at 6, 12, 16 and 24 hours post infection, respectively. These up- and down-regulated DE tags at all four time points post infection in fact represented only 5,028 tags, and included 2,716 DE tags at one, 1,066 at two, 697 at three and 549 at four of four time points, respectively (Table S2). Among them, only 2,319 tags had unique mRNAs and/or genes assigned (Figure 1). After the aforementioned cut-off points for each DE gene were employed 767 tags with mRNA and/or genes assigned remained for further analysis (Figure 1).

**Figure 2:**
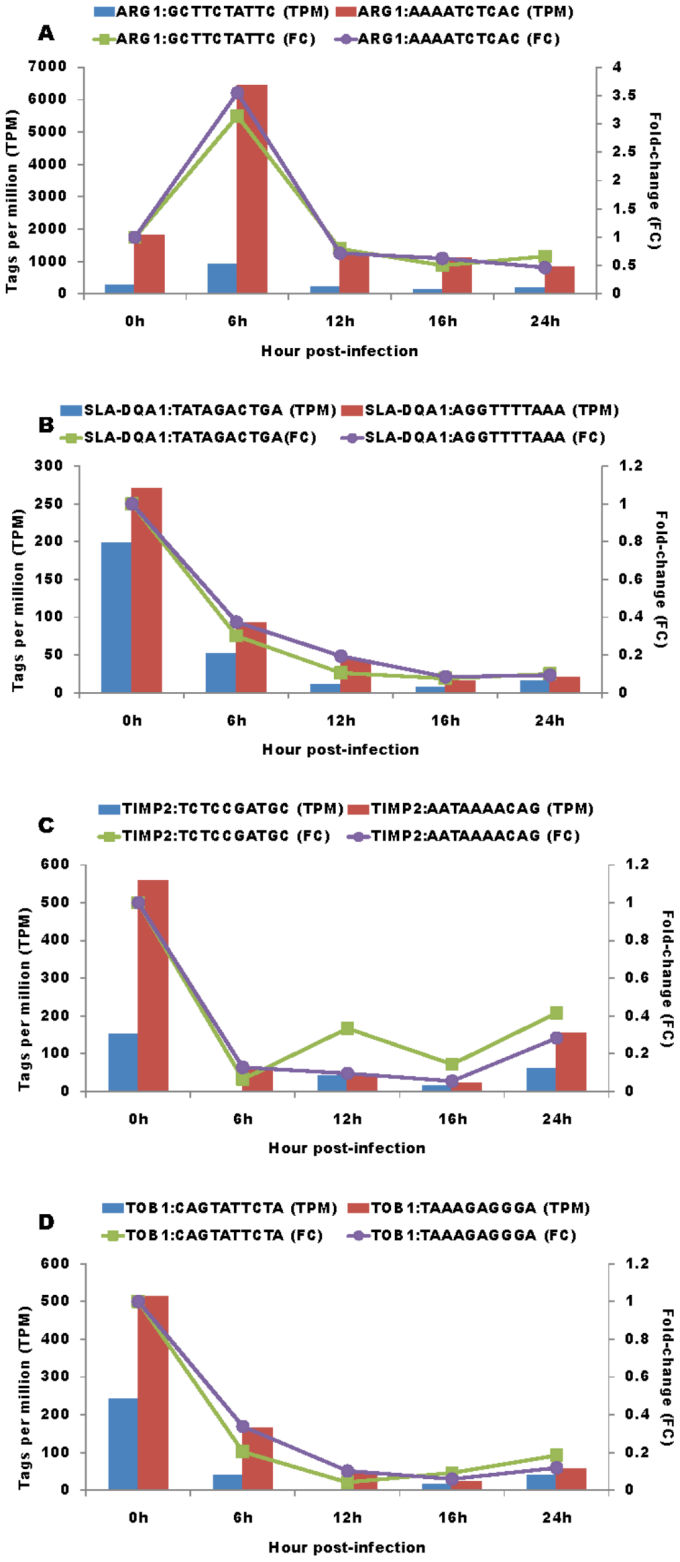
Fold change in TPM for genes with multiple tags due to mRNA isoforms. TPM and fold changes for two tags in *ARG1* (A), *SLA-DQA1* (B), *TIMP2* (C) and *TOB1* (D) representing different mRNA isoforms at 0, 6, 12, 16 and 24 hours post-infection.

**Figure 3:**
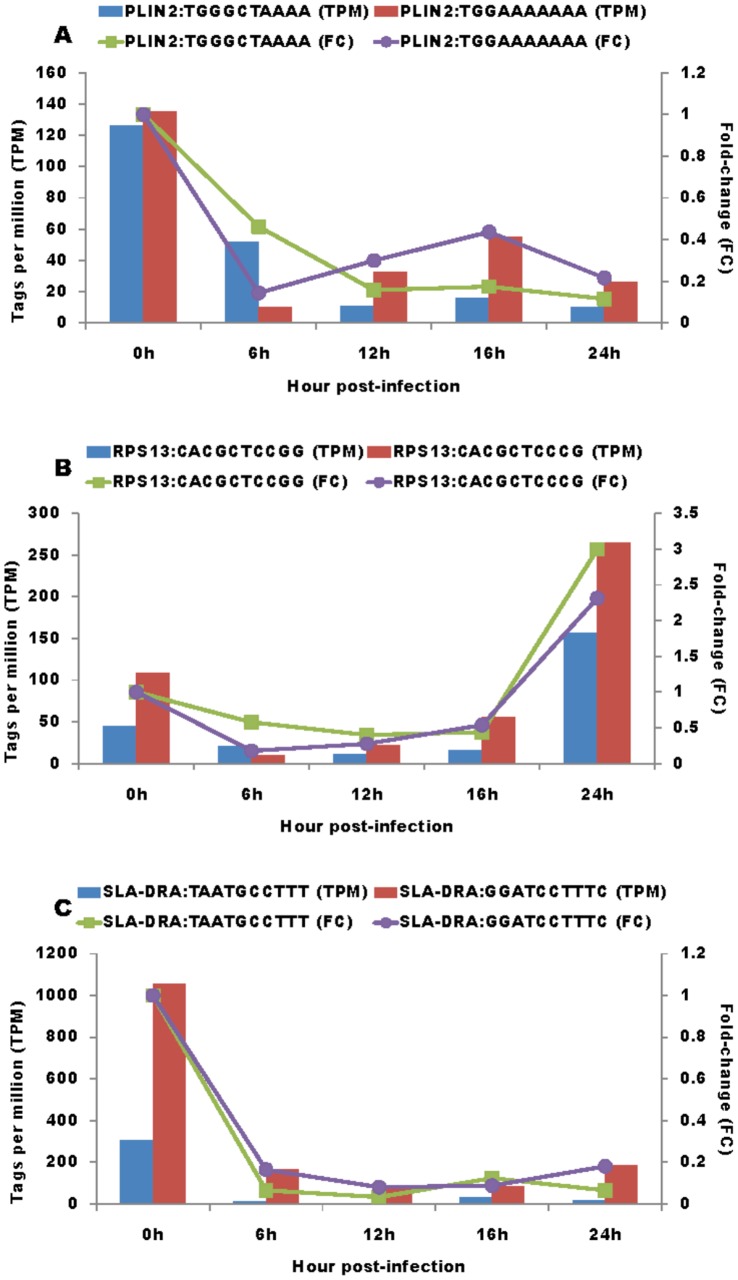
Fold change in TPM for genes with multiple tags due to nucleotide polymorphisms. TPM and fold changes for two tags in *PLIN2* (A), *RPS13* (B) and *SLA-DRA* (C) genes representing different alleles at 0, 6, 12, 16 and 24 hours post-infection.

Manual annotation of these 767 tags with mRNA sequences revealed that they represented a total of 733 genes, and included 700 genes with one tag collected from one unique mRNA sequence, 32 genes with two tags collected from two different mRNA sequences and one gene with three tags collected from three different mRNA sequences, respectively. For those genes that had two or three tags, we further determined whether they represented the true 3′-most tags or not. Interestingly, true cases were confirmed for two tags in *ARG1* (arginase, liver), *SLA-DQA1* (MHC class II, DQ alpha 1), *TIMP2* (TIMP metallopeptidase inhibitor 2) and *TOB1* (transducer of ERBB2, 1) genes (Figure 2A–D) due to different mRNA isoforms and in *PLIN2* (perilipin 2), *RPS13* (ribosomal protein S13) and *SLA-DRA* (MHC class II, DR-alpha) genes (Figure 3A–C) due to nucleotide polymorphisms. Although TPM were variable, trends in fold changes were similar between the two isoforms or two alleles of each gene.

**Figure 4:**
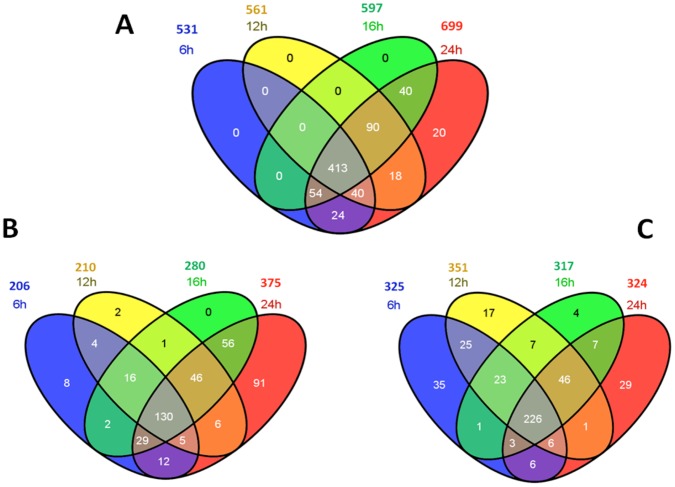
Summary of differentially expressed genes in PAMs infected with PRRSV. All genes (A), up-regulated genes (B) and down-regulated genes at four time-points post-infection (C).

**Figure 5:**
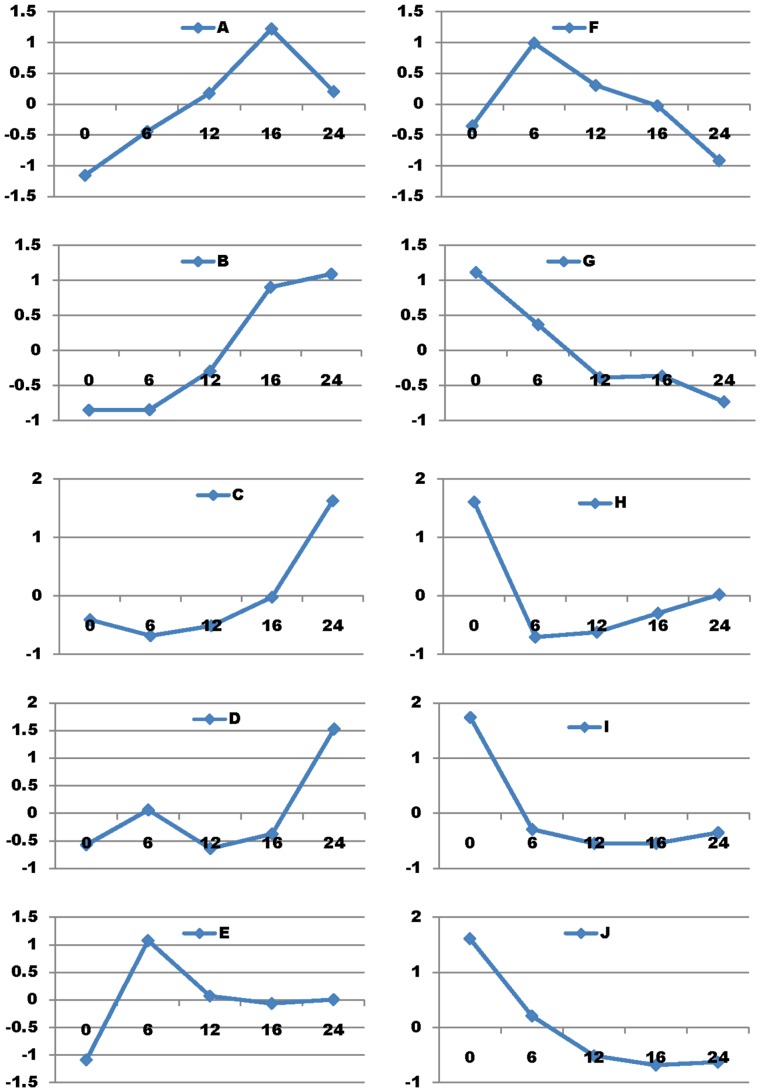
Ten expression trend clusters of 699 DE genes derived from PAMs during PRRSV infection.

Of these 733 pig genes (Figure 1), 699 also had orthologs identified as protein coding genes, while 21 were open reading frame genes (functionally unknown), and one was a non-coding RNA mitochondrial gene in humans. The remaining 12 genes were pig species-specific, including 11 novel genes and a porcine endogenous retrovirus PERV-MSL gene. Except for one novel pig gene (AK351197.1) that was missing both the genomic DNA sequence and location, the rest of the 10 novel genes all had complete genomic DNA sequences with clones mapped to *Sus scrofa* chromosomes (SSCs) 2, 3, 5, 7, 9, 10, 12 and 13, respectively. Compared to the 0 hour mock-infected cells, PRRSV infection induced differential expression of 531, 561, 597, 699 genes (Figure 4A) at 6, 12, 16 and 24 hours post infection, including 206, 210, 280 and 375 genes that were up-regulated (Figure 4B) and 325, 351, 317 and 324 genes that were down-regulated (Figure 4C), respectively at these four time points. Overall, among these 699 DE genes, 226 (63.5%) and 130 (36.5%) were consistently down- or up-regulated, respectively at all four infected time points. Self-organizing map (SOM) method of analysis assigned these 699 DE genes to 10 clusters (Figure 5) based on their expression trends regardless of fold-change magnitudes along these five time points (0 h, 6 h, 12 h, 16 h and 24 h) (Table S3). However, only 573 genes were assigned to pathways, specifically 72 (12.56%) in cluster A, 37 (6.46%) in B, 121 (21.12%) in C, 39 (6.81%) in D, 30 (5.24%) in E, 29 (5.06%) in F, 27 (4.71%) in G, 71 (12.39%) in H, 93 (16.23%) in I and 54 (9.42%) in J.

### Reactome of PAMs Infected with PRRSV: Cellular Processes

**Figure 6:**
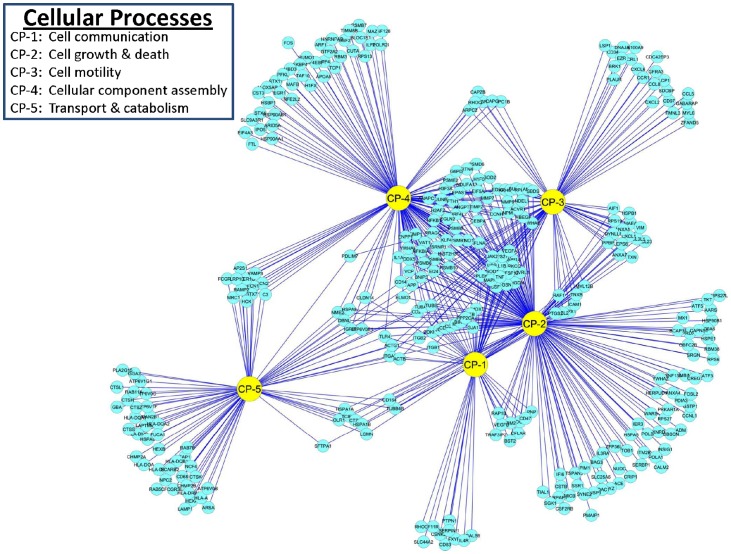
DE gene distributions and interactions among functional categories associated with Cellular Processes in PAMs infected with PRRSV.

The GO, KEGG and REACTOME databases identified 329 DE genes that were involved in cellular processes of PAMs infected with PRRSV (Figure 6). Specific functions included: 1) cell communication, 2) cell growth and death, 3) cell motility, 4) cell organization and biogenesis, and 5) transport and catabolism. Many genes in the system functioned in two or more sub-category pathways; however, there were smaller clusters of genes that contributed to only one cellular process. The largest number of DE genes (191) were broadly involved in cell growth and death and were specifically linked to pathways associated with cell cycle, division, proliferation, growth, cell size regulation, apoptosis, anti-apoptosis, induction and regulation of apoptosis, and regulation of endothelial, fibroblast and smooth muscle cell proliferation. PAMs infected with PRRSV had 153 DE genes that were involved in pathways related to cell organization and biogenesis, which were most notably associated with extracellular matrix organization, macromolecular complex assembly, membrane organization and protein complex assembly, macromolecular/protein complex assembly or disassembly, cellular component biogenesis, organization and size, and macromolecule metabolic/biosynthetic processes. There were 88 DE genes in PRRSV-infected PAMs that are important for cell motility and contributed to pathways related to cell migration, motility, motion and shape; actin cytoskeleton and filament organization; and chemotaxis. Seventy-seven genes important for cellular transport and catabolism were DE in PRRSV-infected PAMs. Most of these DE genes were associated with pathways involved in autophagocytosis, including endocytosis, and lysosomal and phagosomal processes. Cell communication in PAMs infected with PRRSV appears to be quite important because there were 65 DE genes involved in pathways related to cell adhesion, cell junction, cell activation, and cell-cell communication pathways. The 329 DE genes related to cellular process networks of PAMs infected with PRRSV are shown in Figure 6 and are summarized in Table 1.

**Table 1:** Pathway summary of DE genes that are related to six biological systems of PAMs infected with PRRSV.

		Total	Down	Down%	Up	Up%
**Cellular Process**						
Cell Communication	Adhesion - Focal adhesion	14	9	64	5	36
Cell Communication	Adhesion - heterophilic cell adhesion	4	3	75	1	25
Cell Communication	Adhesion - negative regulation of cell adhesion	5	1	20	4	80
Cell Communication	Adhesion - positive regulation of cell adhesion	3	2	67	1	33
Cell Communication	Adhesion - regulation of cell adhesion	7	5	71	2	29
Cell Communication	cell activation - positive regulation of cell activation	13	11	85	2	15
Cell Communication	cell activation - regulation of cell activation	4	1	25	3	75
Cell Communication	Communication - Cell-Cell communication	8	5	63	3	38
Cell Communication	Communication - positive regulation of cell communication	24	16	67	8	33
Cell Communication	Junction - Adherens junction	5	4	80	1	20
Cell Communication	Junction - Cell junction organization	6	5	83	1	17
Cell Communication	Junction - Gap junction	6	4	67	2	33
Cell Communication	Junction - Gap junction trafficking and regulation	5	5	100	0	0
Cell Communication	Junction - Tight junction	9	6	67	3	33
Cell Growth and Death	Apoptosis - anti-apoptosis	34	23	68	11	32
Cell Growth and Death	apoptosis - anti-apoptosis: positive regulation	5	4	80	1	20
Cell Growth and Death	apoptosis - anti-apoptosis: regulation of anti-apoptosis	2	2	100	0	0
Cell Growth and Death	Apoptosis - Apoptotic execution phase	7	2	29	5	71
Cell Growth and Death	Apoptosis - apoptotic mitochondrial changes	7	3	43	4	57
Cell Growth and Death	Apoptosis - apoptotic nuclear changes	4	0	0	4	100
Cell Growth and Death	Apoptosis - negative regulation of apoptosis	36	20	56	16	44
Cell Growth and Death	apoptosis - positive regulation of apoptosis	28	14	50	14	50
Cell Growth and Death	apoptosis - regulation of apoptosis	23	10	43	13	57
Cell Growth and Death	apoptosis - regulation of neuron apoptosis	8	4	50	4	50
Cell Growth and Death	apoptosis and induction of apoptosis	81	39	48	42	52
Cell Growth and Death	Cell cycle - Cell cycle	28	17	61	11	39
Cell Growth and Death	Cell cycle regulation - positive regulation of cell cycle	7	5	71	2	29
Cell Growth and Death	Cell cycle regulation - Regulation of cell cycle	25	12	48	13	52
Cell Growth and Death	Cell cycle, division and proliferation - Meiosis	12	9	75	3	25
Cell Growth and Death	Cell division - positive regulation of cell division	6	2	33	4	67
Cell Growth and Death	Cell division - regulation of cell division	1	1	100	0	0
Cell Growth and Death	cell growth - negative regulation of cell growth	9	4	44	5	56
Cell Growth and Death	cell growth - regulation of cell growth	13	5	38	8	62
Cell Growth and Death	Cell proliferation - cell proliferation	31	17	55	14	45
Cell Growth and Death	Cell proliferation - homeostasis of number of cells	10	3	30	7	70
Cell Growth and Death	Cell proliferation - negative regulation of cell proliferation	50	32	64	18	36
Cell Growth and Death	Cell proliferation - regulation of cell proliferation	5	3	60	2	40
Cell Growth and Death	cell size - regulation of cell size	18	9	50	9	50
Cell Growth and Death	endothelial cell - positive regulation of proliferation	4	2	50	2	50
Cell Growth and Death	fibroblast proliferation - positive regulation	4	2	50	2	50
Cell Growth and Death	fibroblast proliferation - regulation of fibroblast proliferation	1	0	0	1	100
Cell growth and Death	smooth muscle cell - positive regulation of proliferation	6	5	83	1	17
Cell Growth and Death	smooth muscle cell - regulation of proliferation	2	2	100	0	0
Cell Motility	cell migration - positive regulation of cell migration	8	5	63	3	38
Cell Motility	cell migration and motility	24	15	63	9	38
Cell Motility	cell motion	47	28	60	19	40
Cell Motility	cell motion - positive regulation of cell motion	9	5	56	4	44
Cell Motility	cell shape - regulation of cell shape	5	3	60	2	40
Cell Motility	chemotaxis	16	8	50	8	50
Cell Motility	cytoskeleton - actin cytoskeleton organization	30	19	63	11	37
Cell Motility	cytoskeleton - Regulation of actin cytoskeleton	22	13	59	9	41
Cell Motility	filamen - regulation of actin filament depolymerization	5	3	60	2	40
Cell Motility	filament - actin filament organization	7	6	86	1	14
Cell Motility	filament - actin filament-based process	23	16	70	7	30
Cell Motility	filament - regulation of actin filament length	7	3	43	4	57
cell organization and biogenesis	component size - regulation of cellular component size	24	11	46	13	54
cell organization and biogenesis	macromolecular complex assembly	37	19	51	18	49
cell organization and biogenesis	macromolecule - negative regulation of macromolecule biosynthetic/metabolic process	24	15	63	9	38
cell organization and biogenesis	macromolecule - positive regulation of macromolecule biosynthetic/metabolic process	36	19	53	17	47
cell organization and biogenesis	membrane organization	31	10	32	21	68
cell organization and biogenesis	protein complex assembly	31	16	52	15	48
cell organization and biogenesis	macromolecule - regulation of macromolecule biosynthetic/metabolic process	19	9	47	10	53
cell organization and biogenesis	component organization - positive regulation of cellular component organization	18	12	67	6	33
cell organization and biogenesis	component biogenesis - regulation of cellular component biogenesis	16	6	38	10	63
cell organization and biogenesis	component organization - negative regulation of cellular component organization	13	6	46	7	54
cell organization and biogenesis	organelle organization - positive regulation of organelle organization	10	9	90	1	10
cell organization and biogenesis	protein complex - regulation of protein complex assembly	9	2	22	7	78
cell organization and biogenesis	organelle organization - regulation of organelle organization	8	4	50	4	50
cell organization and biogenesis	protein complex - regulation of protein complex disassembly	7	4	57	3	43
cell organization and biogenesis	Extracellular matrix organization	5	3	60	2	40
Transport and Catabolism	endocytosis	26	12	46	14	54
Transport and Catabolism	Lysosome	25	8	32	17	68
Transport and Catabolism	Phagosome	38	21	55	17	45
		1160	**638**		**522**	
**Genetic Information Processing**						
Folding, Sorting and Degradation	Degradation of the extracellular matrix	5	3	60	2	40
Folding, Sorting and Degradation	endopeptidase - regulation of endopeptidase activity	13	9	69	4	31
Folding, Sorting and Degradation	glycosylation - Asparagine N-linked glycosylation	5	3	60	2	40
Folding, Sorting and Degradation	nucleocytoplasmic transport	14	9	64	5	36
Folding, Sorting and Degradation	nucleocytoplasmic transport - positive regulation of nucleocytoplasmic transport	4	4	100	0	0
Folding, Sorting and Degradation	nucleocytoplasmic transport - regulation of nucleocytoplasmic transport	3	2	67	1	33
Folding, Sorting and Degradation	post-Golgi vesicle-mediated transport	7	1	14	6	86
Folding, Sorting and Degradation	proteasomal ubiquitin-dependent protein catabolic process	18	6	33	12	67
Folding, Sorting and Degradation	Protein folding	23	18	78	5	22
Folding, Sorting and Degradation	protein import - regulation of protein import into nucleus	6	5	83	1	17
Folding, Sorting and Degradation	protein import into nucleus	13	9	69	4	31
Folding, Sorting and Degradation	protein localization	52	25	48	27	52
Folding, Sorting and Degradation	protein localization - regulation of protein localization	16	10	63	6	38
Folding, Sorting and Degradation	protein localization in organelle	13	8	62	5	38
Folding, Sorting and Degradation	Protein processing in endoplasmic reticulum	24	18	75	6	25
Folding, Sorting and Degradation	protein targeting	18	9	50	9	50
Folding, Sorting and Degradation	protein transport - intracellular protein transport	68	33	49	35	51
Folding, Sorting and Degradation	protein transport - negative regulation of intracellular Protein transport	13	9	69	4	31
Folding, Sorting and Degradation	Protein transport - regulation of intracellular protein transport	12	9	75	3	25
Folding, Sorting and Degradation	protein ubiquitination - positive regulation of protein ubiquitination	11	2	18	9	82
Folding, Sorting and Degradation	SNARE interactions in vesicular transport	6	1	17	5	83
Replication and Repair	DNA repair	6	3	50	3	50
Replication and Repair	DNA replication	17	10	59	7	41
Replication and Repair	DNA replication - Regulation of DNA replication	11	4	36	7	64
Transcription	DNA binding - negative regulation of DNA binding	6	3	50	3	50
Transcription	DNA binding - positive regulation of DNA binding	9	7	78	2	22
Transcription	DNA binding - regulation of DNA binding	2	2	100	0	0
Transcription	Gene Expression	74	25	34	49	66
Transcription	gene expression - positive regulation of gene expression	33	19	58	14	42
Transcription	gene expression - posttranscriptional regulation of gene expression	24	15	63	9	38
Transcription	mRNA stability	10	6	60	4	40
Transcription	mRNA stability - regulation of mRNA stability	7	5	71	2	29
Transcription	mRNA Stability - Regulation of mRNA Stability by Proteins that Bind AU-rich Elements	17	10	59	7	41
Transcription	NF-kappaB - positive regulation of I-kappaB kinase/NF-kappaB cascade	14	11	79	3	21
Transcription	NF-kappaB - positive regulation of NF-kappaB transcription factor activity	6	6	100	0	0
Transcription	NF-kappaB - regulation of NF-kappaB import into nucleus	4	3	75	1	25
Transcription	Nonsense-Mediated Decay	33	5	15	28	85
Transcription	Processing of Capped Intron-Containing Pre-mRNA	10	7	70	3	30
Transcription	RNA biosynthetic process	18	8	44	10	56
Transcription	RNA Polymerases I, II and III Transcription	7	2	29	5	71
Transcription	Spliceosome	11	11	100	0	0
Transcription	transcription factor - positive regulation of transcription factor activity	8	7	88	1	13
Transcription	transcription factor - regulation of transcription factor import into nucleus	5	4	80	1	20
Transcription	transcription factor - regulation of transcription factor activity	5	3	60	2	40
Translation	Post-translational protein modification	8	5	63	3	38
Translation	ribosomal small subunit biogenesis	5	1	20	4	80
Translation	Ribosome	35	4	11	31	89
Translation	ribosome biogenesis	10	3	30	7	70
Translation	RNA transport	9	4	44	5	56
Translation	SRP-dependent cotranslational protein targeting to membrane	33	3	9	30	91
Translation	translation	53	12	23	41	77
Translation	translation - positive regulation of translation	4	1	25	3	75
Translation	translation - regulation of translation	11	7	64	4	36
Translation	translation elongation	37	6	16	31	84
Translation	Translation Initiation	35	5	14	30	86
Translation	translation initiation - regulation of translational initiation	5	4	80	1	20
Translation	Translation Termination	31	3	10	28	90
		957	**427**	45	**530**	55
**Environmental Information Processing**						
Membrane Transport	Golgi vesicle transport	14	3	21	11	79
Membrane Transport	membrane docking	6	3	50	3	50
Membrane Transport	Membrane Trafficking	16	9	56	7	44
Membrane Transport	secretion - negative regulation of secretion	6	4	67	2	33
Membrane Transport	transport - Aquaporin-mediated transport	3	0	0	3	100
Membrane Transport	transport - SLC-mediated transmembrane transport	7	3	43	4	57
Membrane Transport	transport - Transmembrane transport of small molecules	22	7	32	15	68
Signal Transduction	signal transduction - positive regulation of signal transduction	20	13	65	7	35
Signal Transduction	signal transduction - Ras protein signal transduction	10	3	30	7	70
Signal Transduction	signal transduction - small GTPase mediated signal transduction	21	10	48	11	52
Signal Transduction	signaling - Calcium signaling pathway	7	3	43	4	57
Signal Transduction	signaling - cytokine-mediated signaling pathway	7	5	71	2	29
Signal Transduction	Signaling - ER-nuclear signaling pathway	7	4	57	3	43
Signal Transduction	signaling - platelet-derived growth factor receptor signaling pathway	4	3	75	1	25
Signal Transduction	Signaling by EGFR	8	5	63	3	38
Signal Transduction	Signaling by ErbB	12	9	75	3	25
Signal Transduction	Signaling by FGFR	8	6	75	2	25
Signal Transduction	Signaling by GPCR	30	20	67	10	33
Signal Transduction	Signaling by Jak-STAT	6	3	50	3	50
Signal Transduction	Signaling by MAPK	26	22	85	4	15
Signal Transduction	Signaling by mTOR	5	0	0	5	100
Signal Transduction	Signaling by NGF	20	14	70	6	30
Signal Transduction	Signaling by PDGF	4	2	50	2	50
Signal Transduction	Signaling by SCF-KIT	5	3	60	2	40
Signal Transduction	Signaling by VEGF	9	4	44	5	56
Signal Transduction	Signaling by Wnt	14	7	50	7	50
Signaling Molecules and Interaction	Cell adhesion molecules (CAMs)	15	11	73	4	27
Signaling Molecules and Interaction	cytokine biosynthetic - positive regulation of cytokine biosynthetic process	7	7	100	0	0
Signaling Molecules and Interaction	cytokine biosynthetic - regulation of cytokine biosynthetic process	1	1	100	0	0
Signaling Molecules and Interaction	cytokine production - negative regulation of cytokine production	3	2	67	1	33
Signaling Molecules and Interaction	cytokine production - positive regulation of cytokine production	7	4	57	3	43
Signaling Molecules and Interaction	cytokine production - regulation of cytokine production	7	5	71	2	29
Signaling Molecules and Interaction	Cytokine-cytokine receptor interaction	23	12	52	11	48
Signaling Molecules and Interaction	ECM-receptor interaction	5	2	40	3	60
Signaling Molecules and Interaction	GPCR ligand binding	21	14	67	7	33
		**386**	**223**	58	**163**	42
**Metabolism**						
amide metabolism	cellular amide metabolic process	12	6	50	6	50
Amino Acid Metabolism	Arginine and proline metabolism	5	4	80	1	20
Amino Acid Metabolism	Glutathione metabolism	5	2	40	3	60
Amino Acid Metabolism	Metabolism of amino acids and derivatives	12	3	25	9	75
Biosynthesis of Other Secondary Metabolites	secondary metabolic process	12	5	42	7	58
Carbohydrate Metabolism	alcohol biosynthetic process	10	5	50	5	50
Carbohydrate Metabolism	Amino sugar and nucleotide sugar metabolism	7	4	57	3	43
Carbohydrate Metabolism	carbohydrate biosynthetic process	11	5	45	6	55
Carbohydrate Metabolism	carbohydrate catabolic process	17	4	24	13	76
Carbohydrate Metabolism	catabolic process - negative regulation of catabolic process	4	4	100	0	0
Carbohydrate Metabolism	catabolic process - positive regulation of catabolic process	4	3	75	1	25
Carbohydrate Metabolism	catabolic process - regulation of catabolic process	3	2	67	1	33
Carbohydrate Metabolism	gluconeogenesis	7	4	57	3	43
Carbohydrate Metabolism	glucose import - regulation of glucose import	5	3	60	2	40
Carbohydrate Metabolism	glucose metabolic process	21	6	29	15	71
Carbohydrate Metabolism	glucose transport - negative regulation of glucose transport	4	4	100	0	0
Carbohydrate Metabolism	glucose transport - regulation of glucose transport	2	0	0	2	100
Carbohydrate Metabolism	glutathione metabolic process	5	1	20	4	80
Carbohydrate Metabolism	Glycolysis/Gluconeogenesis	12	2	17	10	83
Carbohydrate Metabolism	Pentose phosphate pathway	7	1	14	6	86
Carbohydrate Metabolism	pentose-phosphate shunt	4	0	0	4	100
Carbohydrate Metabolism	pyruvate metabolic process	10	5	50	5	50
Energy Metabolism	ATP biosynthetic process	13	4	31	9	69
Energy Metabolism	Biological oxidations	9	5	56	4	44
Energy Metabolism	cell redox homeostasis	14	6	43	8	57
Energy Metabolism	cellular respiration	14	2	14	12	86
Energy Metabolism	electron transport chain	18	4	22	14	78
Energy Metabolism	energy coupled proton transport, down electrochemical gradient	9	1	11	8	89
Energy Metabolism	energy derivation by oxidation of organic compounds	15	2	13	13	87
Energy Metabolism	generation of precursor metabolites and energy	45	8	18	37	82
Energy Metabolism	Integration of energy metabolism	8	3	38	5	63
Energy metabolism	mitochondrial ATP synthesis coupled electron transport	10	0	0	10	100
Energy metabolism	mitochondrial electron transport, NADH to ubiquinone	6	0	0	6	100
Energy Metabolism	Mitochondrial Protein Import	5	0	0	5	100
Energy metabolism	mitochondrial transport	7	4	57	3	43
Energy metabolism	mitochondrion organization	13	6	46	7	54
Energy metabolism	monooxygenase - regulation of monooxygenase activity	4	4	100	0	0
Energy metabolism	NAD metabolic process	5	3	60	2	40
Energy Metabolism	nitrogen compound - positive regulation of nitrogen compound metabolic process	40	25	63	15	38
Energy Metabolism	nitrogen compound biosynthetic process	21	10	48	11	52
Energy Metabolism	oxidation reduction	48	13	27	35	73
Energy Metabolism	Oxidative phosphorylation	59	19	32	40	68
Energy metabolism	oxidoreductase - regulation of oxidoreductase activity	5	5	100	0	0
Energy Metabolism	oxygen and reactive oxygen species metabolic process	8	0	0	8	100
Energy metabolism	proton transport	12	2	17	10	83
Energy metabolism	release of cytochrome c from mitochondria	5	2	40	3	60
Energy metabolism	respiratory electron transport chain	12	1	8	11	92
Energy Metabolism	Respiratory electron transport, ATP synthesis by chemiosmotic coupling, and heat production by uncoupling proteins.	23	6	26	17	74
Energy metabolism	respiratory gaseous exchange	6	4	67	2	33
Energy Metabolism	The citric acid (TCA) cycle and respiratory electron transport	27	7	26	20	74
Energy Metabolism	Transport of glucose and other sugars, bile salts and organic acids, metal ions and amine compounds	4	0	0	4	100
Glycan Biosynthesis and Metabolism	hexose metabolic process	24	8	33	16	67
Glycan Biosynthesis and Metabolism	monosaccharide biosynthetic process	9	5	56	4	44
Glycan Biosynthesis and Metabolism	monosaccharide metabolic process	28	11	39	17	61
Glycan Biosynthesis and Metabolism	Other glycan degradation	5	5	100	0	0
Homeostasis	catalytic activity - negative regulation of catalytic activity	27	15	56	12	44
Homeostasis	catalytic activity - positive regulation of catalytic activity	32	16	50	16	50
Homeostasis	homeostasis - calcium ion homeostasis	15	10	67	5	33
Homeostasis	homeostasis - cation homeostasis	22	11	50	11	50
Homeostasis	homeostasis - cellular homeostasis	45	24	53	21	47
Homeostasis	homeostasis - cellular ion homeostasis	30	17	57	13	43
Homeostasis	homeostasis - chemical homeostasis	37	19	51	18	49
Homeostasis	homeostasis - di-, tri-valent inorganic cation homeostasis	21	11	52	10	48
Homeostasis	homeostasis - homeostatic process	61	31	51	30	49
Homeostasis	homeostasis - ion homeostasis	31	18	58	13	42
Homeostasis	homeostasis - iron ion homeostasis	5	1	20	4	80
Homeostasis	homeostasis - multicellular organismal homeostasis	8	3	38	5	63
Homeostasis	hydrolase - negative regulation of hydrolase activity	8	5	63	3	38
Homeostasis	hydrolase - regulation of hydrolase activity	15	12	80	3	20
Homeostasis	molecular function - negative regulation of molecular function	34	19	56	15	44
Homeostasis	molecular function - positive regulation of molecular function	38	20	53	18	47
Homeostasis	phosphate metabolic process	58	25	43	33	57
Homeostasis	phosphorus metabolic process - negative regulation of phosphorus metabolic process	6	6	100	0	0
Lipid Metabolism	Arachidonic acid metabolism	5	3	60	2	40
Lipid Metabolism	carboxylic acid biosynthetic process	12	6	50	6	50
Lipid Metabolism	Fatty acid, triacylglycerol, and ketone body metabolism	8	8	100	0	0
Lipid Metabolism	Glycerophospholipid metabolism	5	2	40	3	60
Lipid Metabolism	Lipid - fatty acid biosynthetic process	8	5	63	3	38
Lipid Metabolism	Lipid - negative regulation of lipid metabolic process	5	4	80	1	20
Lipid Metabolism	Lipid - Regulation of Lipid Metabolism by Peroxisome 2proliferator-activated receptor alpha (PPARalpha)	5	5	100	0	0
Lipid Metabolism	Lipid - Sphingolipid metabolism	4	4	100	0	0
Lipid Metabolism	Lipid - unsaturated fatty acid biosynthetic process	8	6	75	2	25
Lipid Metabolism	lipid localization	12	8	67	4	33
Lipid Metabolism	lipid storage	6	6	100	0	0
Lipid Metabolism	Metabolism of lipids and lipoproteins	21	15	71	6	29
Lipid metabolism	prostaglandin metabolic process	5	4	80	1	20
Lipid Metabolism	Response to elevated platelet cytosolic Ca2+	16	7	44	9	56
Lipid Metabolism	steroid biosynthetic - regulation of steroid biosynthetic process	4	3	75	1	25
Metabolism of Cofactors and Vitamins	coenzyme metabolic process	14	5	36	9	64
Metabolism of Cofactors and Vitamins	cofactor metabolic process	16	6	38	10	63
Metabolism of Cofactors and Vitamins	Metabolism of vitamins and cofactors	7	3	43	4	57
Mineral Metabolism	Iron uptake and transport	9	1	11	8	89
Nucleotides Metabolism	Metabolism of nucleotides	8	4	50	4	50
Nucleotide Metabolism	nucleoside triphosphate catabolic process	4	2	50	2	50
Nucleotide Metabolism	Purine metabolism	7	4	57	3	43
Nucleotide Metabolism	purine nucleoside triphosphate biosynthetic process	14	4	29	10	71
Nucleotide Metabolism	purine nucleotide biosynthetic process	16	6	38	10	63
Nucleotide Metabolism	purine nucleotide metabolic process	19	7	37	12	63
Nucleotide Metabolism	purine ribonucleotide biosynthetic process	15	5	33	10	67
Nucleotide Metabolism	purine ribonucleotide metabolic process	17	5	29	12	71
Nucleotide Metabolism	pyridine nucleotide metabolic process	9	3	33	6	67
Nucleotide Metabolism	Pyrimidine metabolism	5	3	60	2	40
Overview	cellular biosynthetic - positive regulation of cellular biosynthetic process	49	31	63	18	37
Prostanoid Metabolism	Prostanoid metabolism	4	3	75	1	25
Protein Metabolism	Metabolism of proteins	59	15	25	44	75
Protein metabolism	peptidase - negative regulation of peptidase activity	6	3	50	3	50
Protein metabolism	peptidase - regulation of peptidase activity	5	5	100	0	0
Protein metabolism	peptide metabolic process	6	1	17	5	83
Protein metabolism	protein catabolic - regulation of protein catabolic process	7	6	86	1	14
Protein metabolism	protein kinase - positive regulation of protein kinase cascade	16	12	75	4	25
Protein metabolism	protein kinase - regulation of protein kinase cascade	6	3	50	3	50
Protein metabolism	protein metabolic - negative regulation of protein metabolic process	13	10	77	3	23
Protein metabolism	protein metabolic - positive regulation of protein metabolic process	12	7	58	5	42
Protein Metabolism	protein metabolic - regulation of cellular protein metabolic process	2	0	0	2	100
Protein Metabolism	protein metabolic - regulation of protein metabolic process	19	8	42	11	58
Protein metabolism	protein modification - negative regulation of protein modification process	5	4	80	1	20
Protein metabolism	protein modification - positive regulation of protein modification process	5	4	80	1	20
Protein metabolism	protein modification - regulation of protein modification process	14	3	21	11	79
		**1715**	**770**	45	**945**	55
**Organismal Systems**						
Circulatory System	angiogenesis	16	9	56	7	44
Circulatory System	angiogenesis - positive regulation of angiogenesis	5	5	100	0	0
Circulatory System	blood pressure - regulation of blood pressure	10	5	50	5	50
Circulatory System	blood vessel development	20	12	60	8	40
Circulatory System	Cardiac muscle contraction	15	6	40	9	60
Circulatory System	circulatory system process	15	7	47	8	53
Circulatory System	erythrocyte differentiation	6	1	17	5	83
Circulatory System	erythrocyte homeostasis	8	2	25	6	75
Circulatory System	Factors involved in megakaryocyte development and platelet production	9	8	89	1	11
Circulatory System	hemopoiesis	20	10	50	10	50
Circulatory System	Hemostasis	44	28	64	16	36
Circulatory System	Integrin cell surface interactions	6	5	83	1	17
Circulatory System	Muscle contraction	5	2	40	3	60
Circulatory System	myeloid cell differentiation	11	5	45	6	55
Circulatory System	myeloid cell differentiation - negative regulation of myeloid cell differentiation	5	4	80	1	20
Circulatory System	myeloid cell differentiation - regulation of myeloid cell differentiation	4	3	75	1	25
Circulatory System	myeloid leukocyte differentiation - regulation of myeloid leukocyte differentiation	7	5	71	2	29
Circulatory System	Platelet activation, signaling and aggregation	24	12	50	12	50
Circulatory System	Vascular smooth muscle contraction	8	5	63	3	38
Circulatory System	vasoconstriction - regulation of vasoconstriction	5	4	80	1	20
Development	Axon guidance	20	14	70	6	30
Development	cell differentiation - negative regulation of cell differentiation	14	10	71	4	29
Development	cell differentiation - positive regulation of cell differentiation	12	9	75	3	25
Development	cell maturation	8	3	38	5	63
Development	development - positive regulation of developmental process	22	17	77	5	23
Development	Developmental Biology	23	17	74	6	26
Development	developmental growth	9	6	67	3	33
Development	developmental maturation	9	4	44	5	56
Development	mesoderm development	8	4	50	4	50
Development	Osteoclast differentiation	16	13	81	3	19
Development	osteoclast differentiation - regulation of osteoclast differentiation	4	3	75	1	25
Development	Semaphorin interactions	7	5	71	2	29
Development	vasculature development	21	13	62	8	38
Digestive System	Gastric acid secretion	5	5	100	0	0
Digestive System	Mineral absorption	6	3	50	3	50
Digestive System	Pancreatic secretion	6	4	67	2	33
Digestive System	Salivary secretion	5	5	100	0	0
Endocrine System	Adipocytokine signaling pathway	5	5	100	0	0
Endocrine System	Progesterone-mediated oocyte maturation	5	3	60	2	40
Endocrine System	Signaling by GnRH	8	5	63	3	38
Endocrine System	Signaling by insulin	10	4	40	6	60
Endocrine System	Signaling by Insulin receptor	10	1	10	9	90
Endocrine System	Signaling by PPAR	7	5	71	2	29
Environmental Adaptation	hydrogen peroxide metabolic process	5	0	0	5	100
Environmental Adaptation	response to abiotic stimulus	24	16	67	8	33
Environmental Adaptation	response to acid	5	1	20	4	80
Environmental Adaptation	response to amino acid stimulus	4	1	25	3	75
Environmental Adaptation	response to drug	19	12	63	7	37
Environmental Adaptation	response to dsRNA	5	3	60	2	40
Environmental Adaptation	response to endogenous stimulus	34	22	65	12	35
Environmental Adaptation	response to endoplasmic reticulum stress	6	4	67	2	33
Environmental Adaptation	response to ethanol	7	3	43	4	57
Environmental Adaptation	response to external stimulus - positive regulation of response to external stimulus	9	4	44	5	56
Environmental Adaptation	response to external stimulus - regulation of response to external stimulus	4	3	75	1	25
Environmental Adaptation	response to extracellular stimulus	23	13	57	10	43
Environmental Adaptation	response to glucocorticoid stimulus	12	10	83	2	17
Environmental Adaptation	response to heat	6	4	67	2	33
Environmental Adaptation	response to hormone stimulus	30	20	67	10	33
Environmental Adaptation	response to hydrogen peroxide	9	4	44	5	56
Environmental Adaptation	response to hypoxia	17	8	47	9	53
Environmental Adaptation	response to inorganic substance	21	11	52	10	48
Environmental Adaptation	response to insulin stimulus	10	6	60	4	40
Environmental Adaptation	response to mechanical stimulus	7	5	71	2	29
Environmental Adaptation	response to metal ion	12	5	42	7	58
Environmental Adaptation	response to nutrient	14	8	57	6	43
Environmental Adaptation	response to nutrient levels	19	11	58	8	42
Environmental Adaptation	response to organic cyclic substance	10	10	100	0	0
Environmental Adaptation	response to organic nitrogen	7	4	57	3	43
Environmental Adaptation	response to organic substance	68	46	68	22	32
Environmental Adaptation	response to oxidative stress	22	10	45	12	55
Environmental Adaptation	response to oxygen levels	19	9	47	10	53
Environmental Adaptation	response to oxygen radical	4	1	25	3	75
Environmental Adaptation	response to peptide hormone stimulus	13	9	69	4	31
Environmental Adaptation	response to protein stimulus	19	16	84	3	16
Environmental Adaptation	response to reactive oxygen species	11	6	55	5	45
Environmental Adaptation	response to steroid hormone stimulus	17	12	71	5	29
Environmental Adaptation	response to stimulus - positive regulation of response to stimulus	18	11	61	7	39
Environmental Adaptation	response to stress	33	15	45	18	55
Environmental Adaptation	response to temperature stimulus	9	7	78	2	22
Environmental Adaptation	response to unfolded protein	22	17	77	5	23
Environmental Adaptation	response to vitamin	8	5	63	3	38
Excretory System	Collecting duct acid secretion	6	1	17	5	83
Excretory System	Vasopressin-regulated water reabsorption	5	2	40	3	60
Immune System	adaptive immune system	59	38	64	21	36
Immune System	adaptive immune system - positive regulation of adaptive immune response	6	2	33	4	67
Immune System	Cytokine Signaling in Immune system	38	24	63	14	37
Immune System	Cytosolic DNA-sensing pathway	5	3	60	2	40
Immune System	defense response	63	34	54	29	46
Immune System	defense response - positive regulation of defense response	8	4	50	4	50
Immune System	Hematopoietic cell lineage	13	10	77	3	23
Immune System	humoral immune response	11	7	64	4	36
Immune System	IFN - Antiviral mechanism by IFN-stimulated genes	9	4	44	5	56
Immune System	IFN - RIG-I/MDA5 mediated induction of IFN-alpha/beta pathways	9	6	67	3	33
Immune System	IFN - RLR (RIG-like receptor) mediated induction of IFN alpha/beta	5	4	80	1	20
Immune System	immune effector - regulation of immune effector process	11	7	64	4	36
Immune System	immune effector process	14	10	71	4	29
Immune System	Immune System - positive regulation of immune response	23	16	70	7	30
Immune System	immune system development	24	13	54	11	46
Immune System	Immunoregulatory interactions between a Lymphoid and a non-Lymphoid cell	9	6	67	3	33
Immune System	inflammatory response	42	26	62	16	38
Immune System	inflammatory response - acute inflammatory response	11	9	82	2	18
Immune System	inflammatory response - positive regulation of inflammatory response	7	3	43	4	57
Immune System	inflammatory response - regulation of inflammatory response to antigenic stimulus	4	1	25	3	75
Immune System	Innate Immune System	31	18	58	13	42
Immune System	Interferon alpha/beta signaling	10	2	20	8	80
Immune System	Interferon gamma signaling	15	11	73	4	27
Immune System	Interferon Signaling	27	16	59	11	41
Immune System	Interleukin signaling	14	11	79	3	21
Immune System	Intestinal immune network for IgA production	8	8	100	0	0
Immune System	ISG15 antiviral mechanism	9	4	44	5	56
Immune System	L1CAM interactions	12	8	67	4	33
Immune System	leukocyte activation - regulation of leukocyte activation	14	9	64	5	36
Immune System	leukocyte adhesion	7	6	86	1	14
Immune System	leukocyte chemotaxis	5	2	40	3	60
Immune System	leukocyte mediated immunity	10	7	70	3	30
Immune System	leukocyte mediated immunity - positive regulation of leukocyte mediated immunity	5	2	40	3	60
Immune System	leukocyte mediated immunity - regulation of leukocyte mediated immunity	2	2	100	0	0
Immune System	leukocyte migration	19	10	53	9	47
Immune System	leukocyte proliferation - positive regulation of leukocyte proliferation	6	5	83	1	17
Immune System	Leukocyte transendothelial migration	12	7	58	5	42
Immune System	lymphocyte activation - positive regulation of lymphocyte activation	10	8	80	2	20
Immune System	lymphocyte mediated immunity	9	6	67	3	33
Immune System	lymphocyte mediated immunity - regulation of lymphocyte mediated immunity	6	3	50	3	50
Immune System	MAPK targets/Nuclear events mediated by MAP kinases	6	5	83	1	17
Immune System	MyD88 cascade initiated on plasma membrane	13	11	85	2	15
Immune System	MyD88 dependent cascade initiated on endosome	12	10	83	2	17
Immune System	MyD88:Mal cascade initiated on plasma membrane	13	11	85	2	15
Immune System	MyD88-independent cascade initiated on plasma membrane	14	11	79	3	21
Immune System	Natural killer cell mediated cytotoxicity	10	6	60	4	40
Immune System	nitric oxide - positive regulation of nitric oxide biosynthetic process	8	7	88	1	13
Immune System	phagocytosis	7	3	43	4	57
Immune System	phagocytosis - Fc epsilon RI signaling pathway	5	2	40	3	60
Immune System	phagocytosis - Fc gamma R-mediated phagocytosis	8	3	38	5	63
Immune System	response to bacterium	22	11	50	11	50
Immune System	response to lipopolysaccharide	14	8	57	6	43
Immune System	response to molecule of bacterial origin	16	8	50	8	50
Immune System	response to virus	11	3	27	8	73
Immune System	response to wounding	58	35	60	23	40
Immune System	signaling - Chemokine signaling pathway	19	9	47	10	53
Immune System	Signaling - NOD-like receptor signaling pathway	13	10	77	3	23
Immune System	Signaling - Nucleotide-binding domain, leucine rich repeat containing receptor (NLR) signaling pathways	6	4	67	2	33
Immune System	Signaling - Opioid Signalling	5	2	40	3	60
Immune System	signaling - TRIF mediated TLR3 signaling	13	10	77	3	23
Immune System	Signaling by Interleukins	14	11	79	3	21
Immune System	Signaling by RIG-I-like receptor	5	3	60	2	40
Immune System	Signaling by TCR	19	17	89	2	11
Immune System	Signaling by the B Cell Receptor (BCR)	17	9	53	8	47
Immune System	T cell - Antigen processing and presentation	50	27	54	23	46
Immune System	T cell - Costimulation by the CD28 family - T cell	9	9	100	0	0
Immune System	T cell - positive regulation of T cell activation	8	6	75	2	25
Immune System	TAK1 activates NFkB by phosphorylation and activation of IKKs complex	5	5	100	0	0
Immune System	TLR - Innate immune response mediated by toll like receptors	11	7	64	4	36
Immune System	TLR - MAP kinase activation in TLR cascade	9	7	78	2	22
Immune System	TLR - Toll-like receptor signaling pathway	25	15	60	10	40
Immune System	TLR - Trafficking and processing of endosomal TLR	6	1	17	5	83
multicellular organismal process	multicellular organismal - negative regulation of multicellular organismal process	10	5	50	5	50
multicellular organismal process	multicellular organismal - positive regulation of multicellular organismal process	12	7	58	5	42
Nervous System	Cholinergic synapse	5	3	60	2	40
Nervous System	Dopaminergic synapse	6	5	83	1	17
Nervous System	Long-term potentiation	5	4	80	1	20
Nervous System	neurological system - positive regulation of neurological system process	6	5	83	1	17
Nervous System	Neuronal System	11	5	45	6	55
Nervous System	Neurotransmitter Receptor Binding And Downstream Transmission In The Postsynaptic Cell	5	2	40	3	60
Nervous System	Serotonergic synapse	7	3	43	4	57
Nervous System	Signaling - Neurotrophin signaling pathway	13	11	85	2	15
Nervous System	Signaling - NGF signalling via TRKA from the plasma membrane	11	7	64	4	36
Nervous System	synaptic plasticity - regulation of synaptic plasticity	7	6	86	1	14
Nervous System	synaptic transmission - positive regulation of synaptic transmission	6	5	83	1	17
Nervous System	synaptic transmission - regulation of synaptic transmission	5	4	80	1	20
Nervous System	Synaptic vesicle cycle	7	0	0	7	100
Nervous System	Transmission across Chemical Synapses	7	4	57	3	43
Nervous System	vesicle docking during exocytosis	4	1	25	3	75
Nervous System	vesicle-mediated transport	43	13	30	30	70
		**2299**	**1399**	61	**900**	39
**Human Diseases**						
Cancers	Bladder cancer	7	2	29	5	71
Cancers	Glioma	5	3	60	2	40
Cancers	myeloid leukemia - Acute myeloid leukemia	7	4	57	3	43
Cancers	myeloid leukemia - Chronic myeloid leukemia	6	4	67	2	33
Cancers	Pancreatic cancer	6	2	33	4	67
Cancers	Pathways in cancer	22	14	64	8	36
Cancers	Prostate cancer	10	8	80	2	20
Cancers	Renal cell carcinoma	8	3	38	5	63
Cancers	Small cell lung cancer	6	5	83	1	17
Cancers	Transcriptional misregulation in cancer	13	10	77	3	23
Cardiovascular Diseases	Arrhythmogenic right ventricular cardiomyopathy (ARVC)	6	6	100	0	0
Cardiovascular Diseases	Dilated cardiomyopathy	7	6	86	1	14
Cardiovascular Diseases	Hypertrophic cardiomyopathy (HCM)	8	6	75	2	25
Cardiovascular Diseases	Viral myocarditis	11	10	91	1	9
Endocrine and Metabolic Diseases	Diabetes pathways	19	16	84	3	16
Immune Diseases	Allograft rejection	9	8	89	1	11
Immune Diseases	Asthma	9	8	89	1	11
Immune Diseases	Autoimmune thyroid disease	8	7	88	1	13
Immune Diseases	Graft-versus-host disease	11	10	91	1	9
Immune Diseases	Rheumatoid arthritis	32	19	59	13	41
Immune Diseases	Systemic lupus erythematosus	16	16	100	0	0
Infectious Diseases	Amoebiasis	11	10	91	1	9
Infectious Diseases	Bacterial invasion of epithelial cells	7	4	57	3	43
Infectious Diseases	Botulinum neurotoxicity	4	1	25	3	75
Infectious Diseases	Chagas disease (American trypanosomiasis)	17	13	76	4	24
Infectious Diseases	Hepatitis C	11	7	64	4	36
Infectious Diseases	Herpes simplex infection	28	20	71	8	29
Infectious Diseases	HIV Infection	23	4	17	19	83
Infectious Diseases	HTLV-I infection	30	22	73	8	27
Infectious Diseases	Influenza infection	65	28	43	37	57
Infectious Diseases	Legionellosis	18	14	78	4	22
Infectious Diseases	Leishmaniasis	23	21	91	2	9
Infectious Diseases	Malaria	6	4	67	2	33
Infectious Diseases	Measles	16	12	75	4	25
Infectious Diseases	Pathogenic Escherichia coli infection	12	8	67	4	33
Infectious Diseases	Pertussis	19	14	74	5	26
Infectious Diseases	Salmonella infection	17	13	76	4	24
Infectious Diseases	Shigellosis	10	6	60	4	40
Infectious Diseases	Signaling - Epithelial cell signaling in Helicobacter pylori infection	13	5	38	8	62
Infectious Diseases	Staphylococcus aureus infection	13	13	100	0	0
Infectious Diseases	Toxoplasmosis	21	18	86	3	14
Infectious Diseases	Tuberculosis	33	24	73	9	27
Infectious Diseases	Vibrio cholerae infection	10	3	30	7	70
Neurodegenerative Diseases	Alzheimer's disease	30	9	30	21	70
Neurodegenerative Diseases	Amyloids	9	6	67	3	33
Neurodegenerative Diseases	Huntington's disease	33	6	18	27	82
Neurodegenerative Diseases	Parkinson's disease	30	7	23	23	77
Neurodegenerative Diseases	Prion diseases	10	7	70	3	30
		745	**466**	63	**279**	37

A total of 24 pathways in various cellular processes had at least 20 DE genes identified in PAMs infected with PRRSV (Table 1). Of them, PRRSV infection down-regulated more than two thirds of the genes in three pathways: actin filament based processes (69.6%), anti-apoptosis (67.7%) and positive regulation of cell communication (66.7%), while it up-regulated more than two thirds of the genes in two other pathways: membrane organization (67.7%) and lysosome activities (68%) at 24 hours post infection (Table 1).

Among 329 DE genes related to cellular processes, SOM analysis assigned 32 (9.7%), 12 (3.6%), 68 (20.7%), 22 (6.7%), 23 (7.0%), 16 (4.9%), 15 (4.6%), 44 (13.4%), 65 (19.8%) and 32 (9.7%) into expression trend clusters A – J, respectively. All 30 of the following DE genes, *VEGFA*, *ACVRL1*, *GPX1*, *SOD1*, *GSN*, *MAPK1*, *CAPG*, *APP*, *CD24*, *CAPZB*, *UBB*, *LTB*, *PPP2CA*, *IL1B*, *CFL1*, *CDKN1A*, *FLNA*, *TNF*, *ANG*, *EDN1*, *PRKCQ*, *ITGB1*, *JAK2*, *HBEGF*, *HMOX1*, *IL1A*, *NPM1*, *PLEK*, *ACTG1*, *RPS27A* were involved in cellular processes and had multiple functions in at least 10 pathways. The last 17 genes (56.7%) in this list above were clustered in H, I and J, respectively. On the other hand, *CREG1*, *HSBP1*, *H1F0*, *BRK1*, *H1FX*, *CAPG*, *S100A6*, *CAPNS1*, *CD68*, *CTSH*, *MBD3*, *SCARB2*, *FXYD5*, *RNF130*, *TMBIM6*, *LAPTM4A*, *TSPAN31*, *SERPINI1*, *IER3*, *SYNE2*, *CDC42EP3*, *CRIP1*, *ARID5A* and *FMNL3* were exclusively involved in cellular processes: with the first 12 genes (50%) grouped in clusters A, B and C, respectively. A collection of the top 10 up- and bottom 10 down-regulated genes at each time-point post infection made a pool of 19 genes: *TNF*, *HSPA1B*, *TIMP1*, *TNFSF13*, *BAG3*, *HSPA1A*, *ANGPTL4*, *HMOX1*, *GJA1*, *CCRL1*, *HBEGF*, *CCL3L1*, *HSPA6*, *HLA-DOA*, *MAN2B1*, *NUDC*, *HLA-DMB*, *ENPP1* and *PLA2G15* as the most actively down-regulated genes and a pool of 25 genes: *RAB7B*, *IL3RA*, *LRPAP1*, *HLA-A*, *ACVR1*, *ACE*, *CD24*, *MAEA*, *RAB11A*, *SOD2*, *SFTPA1*, *GPX1*, *ARPC2*, *TIAL1*, *H1FX*, *H1F0*, *ATF5*, *MMP9*, *BNIP3*, *LGALS9*, *CCL2*, *CCL8*, *IDO1*, *S100A6*, *CXCL6* as the most actively up-regulated genes in cellular processes.

### Reactome of PAMs Infected with PRRSV: Genetic Information Processing

**Figure 7:**
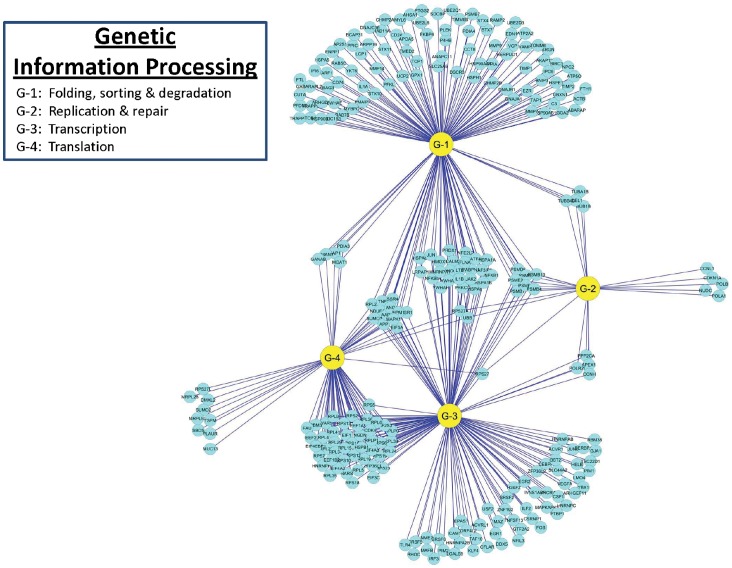
DE gene distributions and interactions among functional categories associated with Genetic Information Processing in PAMs infected with PRRSV.

PRRSV infection of PAMs triggered reactions in 262 genes handling genetic information processing, including transcription, translation, replication and repair, and protein folding, sorting and degradation (Figure 7). Most of the genes involved in genetic information processing were involved in two or more sub-category pathways. However, there were large clusters of genes that functioned exclusively in folding, sorting, and degradation as well as in transcription. In contrast, there were small clusters of genes that only contributed to replication and repair, and translation. There were a total of 147 DE genes related to transcription processes with pathways in DNA binding and regulation, gene expression and regulation, mRNA stability and regulation, regulation of the I-kappaB kinase/NF-kappaB cascades, NF-kappaB transcription factor activity and NF-kappaB import into nucleus, nonsense-mediated decay, processing of capped intron-containing pre-mRNA, RNA biosynthetic processes, RNA polymerases I, II and III transcription, spliceosome and regulation of transcription factors and their import into the nucleus. Protein folding, sorting, and degradation was affected by 147 DE genes with specific functions in degradation of the extracellular matrix, regulation of endopeptidase activity, asparagine N-linked glycosylation, nucleocytoplasmic transport and regulation, post-Golgi vesicle-mediated transport, proteasomal ubiquitin-dependent protein catabolic processes, protein folding, protein import into nucleus and regulation, protein localization and regulation, protein localization in organelles, protein processing in endoplasmic reticulum, protein targeting, intracellular protein transport and regulation, regulation of protein ubiquitination, and Soluble NSF Attachment Protein Receptor (SNARE) interactions in vesicular transport. A role for SNARE machinery in virion egress has been proposed for cytomegalovirus [18] and may be similarly involved with PRRSV egress from PAMs. Seventy-four genes associated with translation processes were DE in PRRSV-infected PAMs and specific pathways were related to post-translational protein modification, ribosome and ribosome biogenesis, RNA transport, signal recognition particle (SRP)-dependent cotranslational protein targeting to membrane, translation and regulation, translation elongation, translation initiation and regulation, and translation termination. Pathways related to repair, replication and regulation were affected by 21 genes that were DE in PAMs infected with PRRSV. The genetic information processing networks of 262 DE genes in PAMs infected with PRRSV are illustrated in Figure 7 and summarized in Table 1.

In the genetic information processing systems, we observed 14 pathways with at least 20 DE genes identified in PAMs infected with PRRSV (Table 1). Among them, more than two-thirds of genes were down-regulated in two pathways, while two-thirds of genes were up-regulated in eight pathways at 24 h post infection. Interestingly, the genes that were down-regulated participated in protein folding (78%) and protein processing in endoplasmic reticulum (75%) pathways and belonged to the broader “protein folding, sorting and degradation” category. The eight up-regulated pathways were related to transcription and translation: transcription processes with gene expression (66%) and nonsense-mediated decay (85%) and translation processes with translation (77%), translation elongation (84%), translation initiation (86%), ribosome (89%), translation termination (90%) and SRP-dependent cotranslational protein targeting to membrane (91%), respectively (Table 1).

For 262 DE genes included in the Genetic Information Processing systems, clusters A – J had 29 (11%), 18 (6.9%), 48 (18%), 19 (7.3%), 14 (5.3%), 13 (5.0%), 13 (5.0%), 31 (12%), 49 (19%) and 28 (11%) genes, respectively. The genes *RPS6*, *RPL23*, *RPS19*, *RPS5*, *RPS16*, *RPS7*, *UBB*, *FLNA*, *TNF*, *NFKBIA*, *JAK2* and *RPS27A* had multiple functions in at least ten pathways with the first six genes (50%) which code for ribosomal proteins clustered in B, C and D, respectively. Twenty one genes: *AKAP12*, *MRPL52*, *SUMO2*, *TSFM*, *MRPL28*, *UBXN1*, *YBX1*, *HELB*, *HNRNPH2*, *AHSA1*, *DSCR3*, *HNRNPC*, *NFIL3*, *PPIC*, *HNRNPA2B1*, *PTBP1*, *DMXL2*, *HNRNPA1*, *LMO4*, *NARS* and *SYNCRIP* had exclusive functions with the last twelve genes (57%) clustered in H, I and J, respectively. The most actively down-regulated genes were *TNF*, *HSPA1B*, *TIMP1*, *TNFSF13*, *BAG3*, *HSPA1A*, *DNAJB1*, *HMOX1*, *GJA1*, *C3*, *NARS*, *FOS*, *EGR1*, *HSPA6*, *YWHAE*, *NUDC*, *ENPP1*, *RAMP2*, *JUNB*, *RPS7* and the most actively up-regulated genes included *CSF1*, *RAB7B*, *CCNH*, *LRPAP1*, *PPIA*, *TRAPPC2*, *NME2*, *MYBPC3*, *ACVR1*, *CD24*, *POLB*, *VEGFA*, *RAB11A*, *YBX1*, *GPX1*, *MRPL28*, *BST2*, *PSME2*, *POLR2I*, *KAP12*, *WARS*, *MMP9*, *BNIP3*, *LGALS9*, *TRAPPC4*, respectively as they appeared either on the top 10 up- or bottom 10 down-regulated genes at least once in PAMs at the four time points post-PRRSV infection.

### Reactome of PAMs Infected with PRRSV: Environmental Information Processing

**Figure 8:**
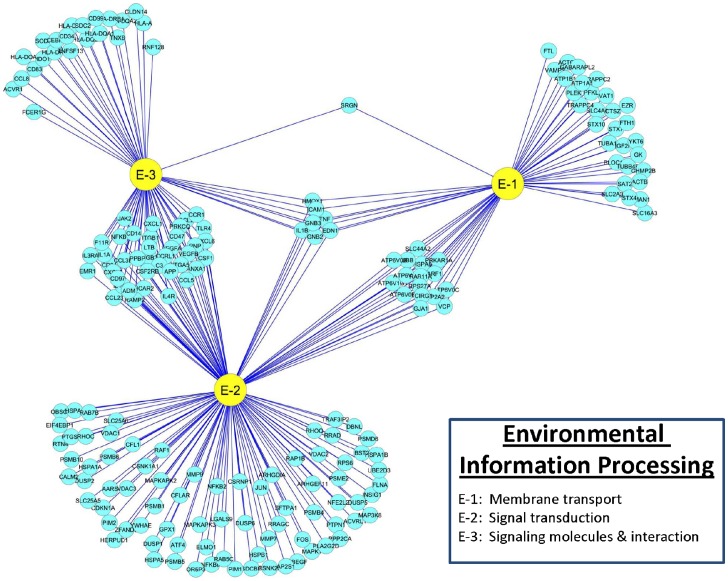
DE gene distributions and interactions among functional categories associated with Environmental Information Processing in PAMs infected with PRRSV.

In the environmental information processing systems, a total of 189 genes differentially expressed in PAMs infected with PRRSV were assigned to three functional categories: 1) membrane transport, 2) signal transduction, and 3) signaling molecules and interaction (Figure 8). While there were large clusters of genes that had exclusive pathway functions, many of the genes involved in environmental information processing contributed to each of the three pathways. The GO, KEGG and REACTOME databases mapped 126 DE genes to functions in signal transduction, such as regulation of signal transduction, Ras protein signal transduction, small GTPase mediated signal transduction, calcium signaling, cytokine-mediated signaling, ER-nuclear signaling, platelet-derived growth factor receptor signaling, and signaling by EGFR, ErbB, FGFR, GPCR, Jak-STAT, MAPK, mTOR, NGF, PDGF, SCF-KIT, VEGF and Wnt, respectively. In addition, 66 DE genes functioned as signaling molecules and interactions, such as cell adhesion molecules, regulation of cytokine biosynthetic processes and production, cytokine-cytokine receptor interaction, ECM-receptor interaction and GPCR ligand binding. Furthermore, 53 DE genes were involved with membrane transport and had functions related to Golgi vesicle transport, membrane docking and trafficking, regulation of secretion, aquaporin-mediated transport, SLC-mediated transmembrane transport, and transmembrane transport of small molecules. The 189 DE genes in PAMs infected with PRRSV involved in environmental information processing networks are illustrated in Figure 8 and summarized in Table 1.

The dominant networks with at least 20 DE genes in the environmental information processing systems included five signal transduction pathways, two signaling molecules and interaction pathways and one membrane transport pathway (Table 1). Among them, at least 65% of the DE genes in PRRSV-infected PAMs at 24 hours post-infection had down-regulation roles in five pathways, including signaling by MAPK (85%), NGF (70%) and GPCR (67%), GPCR ligand binding (67%) and positive regulation of signal transduction (65%). However, genes in only the transmembrane transport of small molecules pathway showed significant up-regulation (68%) by PAMs in response to PRRSV infection 24 hours post-infection (Table 1).

In the environmental information processing systems, expression trend clusters A – J had 22 (12%), 7 (3.7%), 31 (16%), 12 (6.4%), 12 (6.4%), 12 (6.4%), 10 (5.3%), 19 (10%), 44 (23%) and 20 (11%), respectively. The genes *RAF1* and *MAPK1* were involved in 10 and 12 pathways, respectively. The former gene was member of cluster H, while the latter gene belonged to cluster C. Meanwhile, *OR5P3*, *EMR1*, *RRAD* and *HCAR2* were exclusively related to the system and were classified into F, G, I and J clusters, respectively. Compilation of the top 10 up- and bottom 10 down-regulated DE genes in the system each at 6, 12, 16 and 24 hours post infection revealed a pool of 18 genes: *TNF*, *RRAD*, *HSPA1B*, *MAP3K8*, *TNFSF13*, *HSPA1A*, *HMOX1*, *GJA1*, *CCRL1*, *C3*, *HBEGF*, *CCL3L1*, *HSPA6*, *HLA-DOA*, *HLA-DMB*, *RAMP2*, *CD14* and *CTSZ* as the most actively down-regulated genes, while a pool of 20 genes: *RAB7B*, *CD34*, *IL3RA*, *HLA-A*, *ACVR1*, *CD24*, *VEGFA*, *RAB11A*, *GPX1*, *VDAC3*, *PSME2*, *SLC16A3*, *MMP9*, *LGALS9*, *PLA2G2D*, *CCL2*, *CCL8*, *TRAPPC4*, *IDO1* and *CXCL6* as the most actively up-regulated genes, respectively.

### Reactome of PAMs Infected with PRRSV: Metabolisms

**Figure 9:**
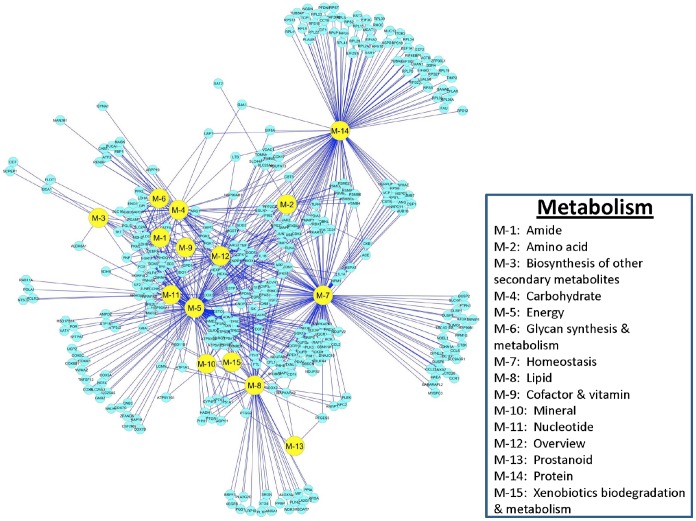
DE gene distributions and interactions among functional categories associated with Metabolism in PAMs infected with PRRSV.

PRRSV infection induced differential expressions of 340 genes in PAMs by 24 hours post-infection that were mainly involved in metabolism of 1) amino acids, 2) carbohydrates, 3) energy, 4) glycans, 5) homeostasis, 6) lipids, 7) cofactors and vitamins, 8) nucleotides and 9) proteins plus a few more functions in amide, secondary metabolites, minerals, prostanoids and cellular biosynthetic processes (Figure 9). The response of the metabolism system of PAMs in response to PRRSV was quite complicated. Small clusters of DE genes identified functioned exclusively in homeostasis, protein metabolism or lipid metabolism. However, the majority of genes were involved in more than two metabolism pathways. More than half (176 genes) of these 340 DE genes were involved in energy metabolism, such as pathways in ATP biosynthetic processes, biological oxidations, cell redox homeostasis, cellular respiration, electron transport chain, energy coupled proton transport, down electrochemical gradient, energy derivation by oxidation of organic compounds, generation of precursor metabolites and energy, integration of energy metabolism, mitochondrial ATP synthesis coupled electron transport, mitochondrial electron transport, NADH to ubiquinone; mitochondrial protein import, mitochondrial transport, mitochondrion organization, regulation of monooxygenase activity, NAD metabolic processes, positive regulation of nitrogen compound metabolic processes, nitrogen compound biosynthetic processes, oxidation reduction, oxidative phosphorylation, regulation of oxidoreductase activity, oxygen and reactive oxygen species metabolic processes, proton transport, release of cytochrome c from mitochondria, respiratory electron transport chain, respiratory electron transport, ATP synthesis by chemiosmotic coupling, heat production by uncoupling proteins, respiratory gaseous exchange, the TCA cycle and respiratory electron transport, and transport of glucose and other sugars, bile salts and organic acids, metal ions and amine compounds. Another group of 151 DE genes participated in homeostasis, such as regulation of catalytic activity, calcium ion homeostasis, cation homeostasis, cellular homeostasis, chemical homeostasis, di- and tri-valent inorganic cation homeostasis, homeostatic processes, ion homeostasis, iron ion homeostasis, multicellular organismal homeostasis, regulation of hydrolase activity, regulation of molecular function and phosphate metabolic processes and regulation. Protein metabolism in PRRSV-infected PAMs was affected by 124 DE genes that were involved in peptidase activity and regulation, peptide metabolic processes and regulation, and regulation of protein kinase cascades, protein metabolic processes and protein modification processes. The data analysis also revealed 64, 48 and 49 DE genes having functions in metabolism of lipids, carbohydrates, and positive regulation of cellular biosynthetic processes, respectively. The remaining metabolic categories had 35 and fewer DE genes involved. The metabolism networks of 340 DE genes in PAMs infected with PRRSV are shown in Figure 9 and summarized in Table 1.

Among pathways involved in energy metabolism, genes related to generation of precursor metabolites and energy; the TCA cycle and respiratory electron transport; respiratory electron transport, ATP synthesis by chemiosmotic coupling and heat production by uncoupling proteins; oxidation reduction; and oxidative phosphorylation accounted for 82%, 74%, 74%, 73% and 68% of the up-regulated DE genes, respectively (Table 1). In homeostasis, 11 pathways had more than 20 DE genes identified, but none of these homeostasis pathways had two-thirds of the DE genes either down- or up-regulated. Other important pathways needing to be mentioned in the metabolism systems include: metabolism of proteins (59 DE genes with 44 (75%) up-regulated), hexose metabolic processes (24 DE genes with 16 genes (67%) up-regulated), metabolism of lipids and lipoproteins (21 genes with 15 (71%) down-regulated) and glucose metabolic processes (21 DE genes with 15 (71%) up-regulated), respectively (Table 1).

Of the 340 DE genes that were involved in the metabolism systems in PRRSV-infected PAMs, 46 (14%) were assigned to cluster A, 25 (7.4%) to B, 74 (22%) to C, 26 (7.7%) to D, 18 (5.3%) to E, 15 (4.4%) to F, 14 (4.1%) to G, 44 (13%) to H, 46 (14%) to I and 32 (9.4%) to J, respectively. The following 55 genes in the metabolism systems had multiple functions in at least 10 pathways: *SOD2*, *SOD1*, *GPX1*, *ATP5C1*, *ATP6V0B*, *NME2*, *NDUFB3*, *NDUFS2*, *NDUFV2*, *FTL*, *NDUFAB1*, *ATP5J2*, *PGLS*, *UQCR10*, *FTH1*, *G6PD*, *ATP6V0E1*, *TPI1*, *COX3*, *MDH2*, *ATP6V1F*, *GPI*, *ATP5G2*, *NDUFB2*, *TALDO1*, *IFI6*, *UQCR11*, *CCL2*, *APP*, *TCIRG1*, *ATP6V0C*, *COX2*, *NDUFA4*, *CD24*, *HEXA*, *TXNRD1*, *PNP*, *ATP5O*, *APOA5*, *ATF4*, *IL1B*, *GPD1*, *ENPP1*, *LDHB*, *JUN*, *SDHB*, *FBP1*, *TNF*, *EDN1*, *JAK2*, *HEXB*, *ATP2A2*, *HMOX1*, *HERPUD1* and *ADM* with the first 28 genes (50.91%) clustered in A, B and C, respectively, while the last 15 genes (27.27%) in H and I clusters, respectively. The metabolism systems specific genes were *SDS*, *PGAM1*, *GBE1*, *CYP51A1*, *HSD11B1*, *ENOPH1*, *NADH5*, *PTGR1*, *DDT*, *PPM1G*, *TBC1D1*, *ATP5J2*, *PGLS*, *ISYNA1*, *PHYH*, *HSD17B14*, *MBOAT7*, *PGS1*, *TPI1*, *MDH2*, *TALDO1*, *PGK1*, *COX17*, *CNDP2*, *SH3BGRL3*, *BCKDK*, *NAGK*, *POR*, *GLRX*, *SLC25A3*, *ISCA1*, *AMPD2*, *ALDH8A1*, *PTGES3*, *TXNL1*, *GSTO1*, *GPD1*, *LDHB*, *CYP4F3*, *HADH*, *NT5C2*, *SCPEP1*, *CKB*, *RIOK3*, *TBC1D10A*, *TBC1D20* with the first 28 genes (61%, 28/46) grouped into clusters A, B and C, respectively. Two sets of DE genes, one with *TNF*, *TIMP1*, *MAP3K8*, *TNFSF13*, *ANGPTL4*, *HMOX1*, *GJA1*, *NDEL1*, *PMAIP1*, *FOS*, *DUSP6*, *ANG*, *EGR1*, *MGST1*, *PLAUR*, *MAN2B1*, *YWHAE*, *ENPP1*, *JUNB*, *HSPB1*, *ARSA*, *PLA2G15* and *RPS7* and the other with *CSF1*, *HEXA*, *LRPAP1*, *PPIA*, *MYBPC3*, *ACVR1*, *ACE*, *CD24*, *MAEA*, *RAB11A*, *SOD2*, *SFTPA1*, *GPX1*, *HSD11B1*, *PSME2*, *POLR2I*, *TBC1D1*, *BCKDK*, *SLC16A3*, *BNIP3*, *LGALS9*, *PLA2G2D*, *CCL2*, *SDS* and *IDO1* were identified as the most actively down- and up-regulated genes, respectively in the metabolism system.

### Reactome of PAMs Infected with PRRSV: Organismal Systems

**Figure 10:**
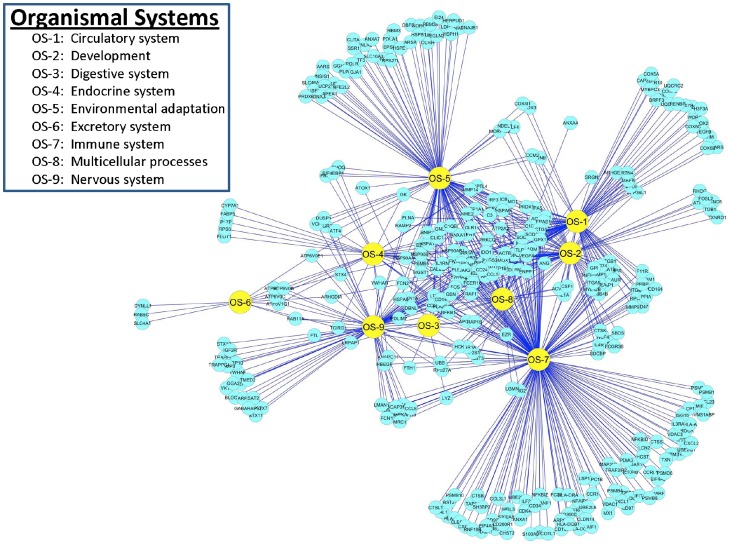
DE gene distributions and interactions among functional categories associated with Organismal Systems in PAMs infected with PRRSV.

A total of 346 DE genes identified in PAMs infected with PRRSV impacted organismal systems, shown in Figure 10 and summarized in Table 1. All of the pathways involved in organismal systems were affected by genes that functioned in multiple processes. There were 30 pathways in organismal systems of PAMs infected with PRRSV at 24 hours post infection that had 20 or more DE genes (Table 1). More than two-thirds of DE genes were down-regulated in eight of these pathways - positive regulation of developmental processes (77%), response to unfolded protein (77%), developmental biology (74%), axon guidance (70%), positive regulation of immune response (70%), response to organic substance (68%), response to hormone stimulus (67%) and response to abiotic stimulus (67%), In comparison, more than two-thirds (70%) of the DE genes in the vesicle docking during exocytosis pathway were up-regulated (Table 1).

The immune system held the largest group of 297 DE genes that included genes involved in adaptive immunity and regulation, cytokine signaling, cytosolic DNA-sensing pathways, defense response and regulation, hematopoietic cell lineage, humoral immune response, antiviral mechanisms by IFN-stimulated genes, RIG-I/MDA5 mediated induction of IFN-α/β pathways, RLR (RIG-like receptor) mediated induction of IFN-α/β, immune effector processes and regulation, regulation of immune response, immune system development, immunoregulatory interactions between a lymphoid and a non-lymphoid cell, inflammatory response and regulation, acute inflammatory response, regulation of inflammatory response to antigenic stimulus, innate immune system, interferon α/β signaling, IFN-γ signaling, IFN signaling, interleukin signaling, intestinal immune network for IgA production, ISG15 antiviral mechanism, L1CAM interactions, regulation of leukocyte activation, leukocyte adhesion, leukocyte chemotaxis, leukocyte mediated immunity and regulation, regulation of leukocyte mediated immunity, leukocyte migration, positive regulation of leukocyte proliferation, leukocyte transendothelial migration, regulation of lymphocyte activation, lymphocyte mediated immunity and regulation, MAPK targets/nuclear events mediated by MAP kinases, MyD88 cascades initiated on plasma membrane, MyD88 dependent cascades initiated on endosome, MyD88:Mal cascades initiated on plasma membranes, MyD88-independent cascades initiated on plasma membrane, natural killer cell mediated cytotoxicity, regulation of nitric oxide biosynthetic processes, phagocytosis, Fc-ε RI signaling pathway, Fc-γ R-mediated phagocytosis, response to bacterium, response to lipopolysaccharide, response to molecule of bacterial origin, response to virus, response to wounding, chemokine signaling pathway, NOD-like receptor signaling pathway, nucleotide-binding domain, leucine rich repeat containing receptor signaling pathways, opioid signaling, TRIF mediated TLR3 signaling, signaling by interleukins, signaling by RIG-I-like receptor, signaling by TCR, signaling by the B cell receptor, antigen processing and presentation, stimulation by the CD28 family, regulation of T cell activation, TAK1 activation of NFκB by phosphorylation and activation of IKKs complex, innate immune response mediated by toll like receptors, MAP kinase activation in TLR cascades, toll-like receptor signaling pathways and trafficking and processing of endosomal TLR.

### Reactome of PAMs Infected with PRRSV: Human Diseases

**Figure 11:**
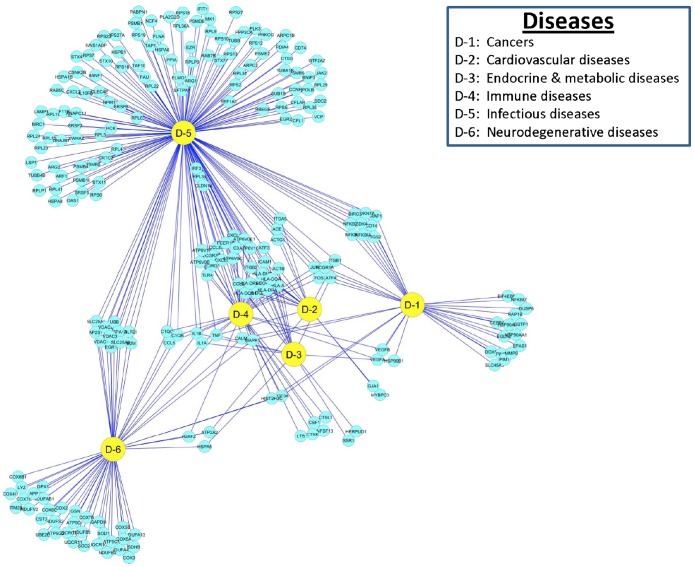
DE gene distributions and interactions among functional categories associated with Human Diseases in PAMs infected with PRRSV.

As a disease in pigs, PRRSV infection of PAMs caused DE of 234 genes that share pathways associated with human diseases, such as 1) cancers, 2) cardiovascular diseases, 3) endocrine and metabolic diseases, 4) immune diseases, 5) infectious diseases and, 6) neurodegenerative diseases (Figure 11; Table 1). Interestingly, most genes had exclusive functions in each disease subcategory. Among them, 169 DE genes are important in human infectious diseases, including pathways for amoebiasis, bacterial invasion of epithelial cells, botulinum neurotoxicity, Chagas disease (American trypanosomiasis), hepatitis C, herpes simplex infection, HIV infection, HTLV-I infection, influenza infection, legionellosis, leishmaniasis, malaria, measles, pathogenic Escherichia coli infection, pertussis, *Salmonella* infection, shigellosis, Signaling - Epithelial cell signaling in *Helicobacter pylori* infection, *Staphylococcus aureus* infection, toxoplasmosis, tuberculosis and *Vibrio cholerae* infection (Table 1). Fifty-seven genes important in the courses of five human neurodegenerative diseases - Alzheimer's disease, Amyloids, Huntington's disease, Parkinson's disease and prion diseases were also DE in PRRSV-infected PAMs. A total of 42 DE genes expressed in PAMs infected with PRRSV were also involved in human immune diseases, such as allograft rejection, asthma, autoimmune thyroid disease, graft-versus-host disease, rheumatoid arthritis and systemic lupus erythematosus. In addition, KEGG and REACTOME pathway analyses revealed 36, 19 and 18 genes that are related to human cancer diseases, endocrine and metabolic diseases and cardiovascular diseases (Table 1) are also DE in PAMs after infection with PPRSV. The Human Disease networks shared with PRRSV-infected PAMs are illustrated in Figure 11.

PAMs infected with PRRSV had more than 20 DE genes that are involved in the gene expression pathways of seven human infectious diseases, three neurodegenerative diseases, one immune disease and one pathway in cancer (Table 1). More than two-thirds of the genes associated with leishmaniasis (91%), toxoplasmosis (86%), HTLV-I infection (73%), tuberculosis (73%) and herpes simplex infection (71%), were down-regulated in PAMs infected with PRRSV. On the other hand, PRRSV infection of PAMs up-regulated more than 66% of the genes commonly associated with four human diseases/pathways, including HIV infection (83%), Huntington's disease (82%), Parkinson's disease (77%) and Alzheimer's disease (70%), respectively.

Cluster analysis showed clusters A – J contained 25 (11%), 14 (6.0%), 54 (23%), 23 (10%), 8 (3.4%), 9 (3.9%), 10 (4.3%), 28 (12%), 47 (20%) and 16 (6.8%), respectively, of the 234 genes that were DE in human disease pathways of PRRSV-infected PAMs. Nineteen of the DE genes were involved in at least 10 human disease pathways and included *MAPK1*, *TLR4*, *IL1B*, *HLA-DMB*, *RAF1*, *JUN*, *ACTB*, *TNF*, *HLA-DRA*, *HLA-DRB1*, *HLA-DOA*, *HLA-DQA2*, *HLA-DQB1*, *NFKBIA*, *ITGB1*, *IL1A*, *NFKB1*, *HLA-DQA1* and *ACTG1* with the last 17 (89%) clustered in H, I and J, respectively. Among six systems in human diseases, *SLC45A3*, *CLEC4E* and *CRTC2* were exclusively involved in human disease network pathways. A set of 18 genes: *C1QB*, *C3*, *CCL3L1*, *CD14*, *DNAJB1*, *DUSP6*, *EGR1*, *FOS*, *GJA1*, *HBEGF*, *HLA-DMB*, *HLA-DOA*, *HSPA1A*, *HSPA1B*, *HSPA6*, *RPS7*, *TNF*, *TNFSF13* and a set of 25 genes: *ACE*, *ARPC2*, *BNIP3*, *CCL2*, *CCNH*, *CSF1*, *CTSK*, *CXCL6*, *GPX1*, *HLA-A*, *IFIT1*, *MMP9*, *MX1*, *MYBPC3*, *OAS1*, *PLA2G2D*, *POLB*, *POLR2I*, *PPIA*, *PSME2*, *RAB7B*, *SFTPA1*, *SOD2*, *VDAC3* and *VEGFA* formed a pool of genes that were predominantly down and up- regulated, respectively in the five categories of human diseases.

## Discussion

In the present study, using 91,807 unique tags derived from five SAGE libraries collected from 0 hour mock-infected and 6, 12, 16 and 24 hours PRRSV-infected PAM cells, we identified a total of 699 functionally known genes that showed at least 2.0 fold changes in expression at one of the first three post-infection time points (6, 12 and 16 hours) and were at least 1.5 fold different at the 24 hours post-infection compared to the 0 hour mock infected cells. Our transcriptome profiling represents the largest known set of DE genes of PAMs challenged with PRRSV. The list of DE genes found in our present investigation was extensive, but many were unique as we found that only 8 of 108 (7.4%), 50 of 215 (23%) and 47 of 294 (16%) known coding genes previously reported [19]–[21] were also DE in PRRSV-infected PAMs in our study. Genini and colleagues [19] performed an *in vitro* study using PAMs obtained from six piglets and challenged with the Lelystad PRRSV strain. The European Lelystad strain of PRRSV has biological similarities but distinct serological properties from the North American VR-2332 isolate [22]. Gene expression was investigated using Affymetrix microarrays, but with very limited annotation available at that time. They detected a total of 1,409 differentially expressed transcripts based on analysis of variance, and found two, five, 25, 16 and 100 transcripts that differed from controls by a minimum of 1.5-fold at 1, 3, 6, 9 and 12 h post-infection, respectively. In addition to three uninfected negative controls, Xiao et al. [20] challenged six conventionally-reared, healthy 6-week-old, crossbred weaned pigs (Landrace×Yorkshire) with the classical North American type PRRSV (N-PRRSV) strain CH 1a. Lung tissues were collected from the control group, three pigs at 96 h (N96) and three pigs at 168 h (N168) post infection and mRNA was extracted. Transcriptome profiling was performed using a Solexa/Illumina next generation sequencing method. Although the authors claimed that there were 5,430 DE genes between all time points (N96/C, N168/C, N168/N96) during infection, they only assigned 215 DE genes to pathways. Using Affymetrix microarrays, Zhou and co-workers [21] reported 294 functionally known genes that were differentially expressed in PAMs derived from three uninfected and three infected 5-week-old Tongcheng pigs at 5 days post infection. The infected groups were challenged with PRRSV-WUH3 by intramuscular inoculation. Here, we performed an *in vitro* study on PAMs challenged with PRRSV strain VR-2332 and carried out the transcriptome analysis using the SAGE technology. Collectively, these investigations examined responses to infections with different PRRSV strains using either *in vitro* or *in vivo* approaches and time courses ranged from 0 to 168 hours. In addition, two different types of tissues, PAMs or lung tissue, were collected for transcriptome profiling using either microarray or tag-based sequencing. Therefore, different experimental designs, transcription profiling formats, time course ranges, virus strains and tissue sources are all reasons that explain the low incidence of common DE genes among the different investigations.

Direct sequencing (whole transcriptome shotgun sequencing or RNA-seq) is likely to yield similar results to SAGE. The Illumina sequencing techniques usually produce sequences with a maximum of 100 bp in length. The major drawbacks of whole transcriptome shotgun sequencing or RNA-seq include insufficient detection of genes/transcripts with low levels of expression, uneven sequencing depth along the length of a transcript and impossible usage of spreadsheet software for data processing due to large file size, (as reviewed by [23]).

Three databases: DAVID (The Database for Annotation, Visualization and Integrated Discovery, v6.7, http://david.abcc.ncifcrf.gov/home.jsp), KEGG (Kyoto Encyclopedia of Genes and Genomes, http://www.genome.jp/kegg/pathway.html) and REACTOME (http://www.reactome.org/ReactomeGWT/entrypoint.html) were used in the present study to assign DE genes in PAMs infected with PRRSV to functional pathways. The DAVID Bioinformatics database is owned by the NIH. The team developed a unique single linkage method by which >20 gene identifier types and >40 functional annotation categories from dozens of heterogeneous public databases have been comprehensively integrated in the DAVID Knowledgebase [24]. The KEGG database is described as a resource for understanding high-level functions and utilities of biological systems, such as the cell, the organism and the ecosystem, from molecular-level information, especially large-scale molecular datasets generated by genome sequencing and other high-throughput experimental technologies. The REACTOME tool claims to host a manually curated and peer-reviewed pathway database with cross references to many bioinformatics databases, such as NCBI Entrez Gene, Ensembl and UniProt databases, the UCSC and HapMap Genome Browsers, the KEGG Compound and ChEBI small molecule databases, PubMed, and Gene Ontology. Initially, the DAVID, KEGG and REACTOME databases helped us assign 517, 383 and 369 of 699 DE genes, respectively, to functional pathways. We then combined pathways generated by these three databases and classified them into six systems including 1) cellular processes, 2) genetic information processing, 3) environmental information processing, 4) metabolism, 5) organismal systems and 6) human diseases based on the KEGG classification systems with modifications. Merging the same/similar pathways and editing the overlapping pathways led to functional classification of 573 DE genes. These processes indicate that combining pathway information from different databases helps maximize the coverage of DE genes in pathway analysis.

As shown in Figures 6–11 and Table 1, each of these six systems described above has several functional categories ranging from three in environmental information processing to fifteen in metabolism. The category with the greatest number of DE genes in each of these six systems belongs to the cell growth and death with 191 DE genes identified in cellular processes, the transcription processes with 147 DE genes in the genetic information processing, signal transductions with 126 DE genes in the environmental information processing, energy metabolism with 176 DE genes in metabolism, the immune system with 297 DE genes in organismal systems, and infectious diseases in human disease. These pathway categories with the most DE genes clearly confirmed the basic characteristics of PAMs in pigs responding to PRRSV infection reported by other researchers. For example, Costers and co-workers [25] found that PRRSV stimulates anti-apoptotic pathways in PAMs early in infection and the PRRSV-infected macrophages die by apoptosis late in infection. Gudmundsdottir and Risatti [26] investigated the effect of PRRSV infection on activation of 25 immunomodulatory cellular genes in PAMs at 24 and 48h post-infection and found a regulatory role of PRRSV ORF1A on PAM gene expression. During virus infection, PRRSV modulates the transcription and translation of the host cell to make them survive and propagate [27]. PRRSV infection also suppresses gene expression. Using two-dimensional liquid chromatography-tandem mass spectrometry coupled with isobaric tags for relative and absolute quantification (iTRAQ) labeling approach, Lu and colleagues [28] just recently revealed that signal transduction is one of the differentially expressed proteome components in PAMs infected with PRRSV. Research has shown that viruses and other pathogens usually slow down the host cell’s energy production in order to enhance infection [29], [30]. Resistance response is an expensive activity, which would consume a large amount of energy. Appropriate energy management is important to restrict pathogen propagation or to repair the cells [31]. Innate immunity is critical to the host for defense against various pathogens. PRRSV infection under certain circumstances fails to elicit some components of the innate response [32]. Adaptive immune response is also important to kill viruses. Adaptive immunity depends on antigen presentation, where the MHC (major histocompatibility complex) class II molecule binds antigen to trigger an appropriate adaptive immune response and restrict pathogen growth [33]. We are the first to report DE genes that are common between PRRSV infection and 22 human infectious diseases. In particular, PRRSV infection of PAMs induced DE of 65, 33, 30, 28, 23, 23, and 21 genes that are commonly associated with influenza infection, tuberculosis, HTLV-I infection, Herpes simplex infection HIV Infection, leishmaniasis, and toxoplasmosis, respectively.

A dominant pathway was defined as a pathway with 20 or more DE genes identified in the present study. The numbers of dominant pathways with more than two-thirds of DE genes down- or up-regulated in PAMs infected with PRRSV at 24 h post infection showed some interesting, but different trends among the six systems described above. In the cellular processes and the human diseases systems, the numbers of dominant pathways were relatively even: three down- vs. two up-regulated in the former case and five down- vs. four up-regulated in the latter case. However, in the genetic information processing system there were two down- vs. eight up-regulated pathways and one down- vs. eight up-regulated pathways in the metabolism systems. In contrast, the down- to up-regulated pathway ratio was 5∶1 in the environmental information processing systems and 8∶1 in organismal systems. The dominant pathways with two-thirds of DE genes down-regulated in PAMs infected with PRRSV at 24 h post infection were: actin filament based processes (70%), anti-apoptosis (68%) and positive regulation of cell communication (67%) in cellular processes; protein folding (78%) and protein processing in endoplasmic reticulum (75%) in the genetic information processing systems; signaling by MAPK (85%), NGF (70%) and GPCR (67%), GPCR ligand binding (67%) and positive regulation of signal transduction (65%) in the environmental information processing systems; metabolism of lipids and lipoproteins (71%) in the metabolism systems; positive regulation of developmental processes (77%), response to unfolded protein (77%), developmental biology (74%), axon guidance (70%), positive regulation of immune response (70%), response to organic substances (68%), response to hormone stimulus (67%) and response to abiotic stimulus (67%) in organismal systems; and leishmaniasis (91%), toxoplasmosis (86%), HTLV-I infection (73%), tuberculosis (73%) and Herpes simplex infection (71%) in the human diseases system (Table 1). The dominant pathways with two-thirds of DE genes up-regulated in PAMs infected with PRRSV at 24 h post infection included: membrane organization (68%) and lysosome (68%) in cellular processes; gene expression (66%), nonsense-mediated decay (85%), translation (77%), translation elongation (84%), translation initiation (86%), ribosome (89%), translation termination (90%) and SRP-dependent cotranslational protein targeting to membrane (91%) in the genetic information processing systems; transmembrane transport of small molecules (68%) in the environmental information processing systems; generation of precursor metabolites and energy (82%), the citric acid cycle and respiratory electron transport (74%), respiratory electron transport (74%), ATP synthesis by chemiosmotic coupling, and heat production by uncoupling proteins (73%), oxidation reduction and oxidative phosphorylation (68%), metabolism of proteins (75%), hexose metabolic processes (67%) and glucose metabolic processes (71%) in the metabolism systems; vesicle docking during exocytosis (70%) in the organismal systems; and HIV Infection (83%), Huntington's disease (82%), Parkinson's disease (77% and Alzheimer's disease (70%) in the human diseases system (Table 1).

As shown in Figure 5, we classified these 699 DE genes into ten clusters based on their expression trends. The abundance of DE genes in cluster A increased from initial infection until 16 h post-infection. Thereafter, gene abundances decreased, but remained up-regulated at 24 h post infection. In comparison, DE gene abundances in Cluster B did not change between 0 h and 6 h post-infection, but rapidly increased by 16 h, and increased slightly at 24 h post infection. The relative abundances of DE genes in cluster C decreased to their lowest levels at 6 h, but gradually increased to reach their highest levels at 24 h post infection. The DE genes in cluster D were up-regulated at 6 h, returned to pre-infection levels between 12 h and 16 h, and were dramatically up-regulated at 24 h post infection. Abundances of DE genes in both clusters H and I decreased dramatically by 6 h post-infection. Expression levels of genes in cluster H slowly returned to pre-infection amounts by 24 h post-infection, while gene abundances in cluster I remained at similar down-regulated levels between 6 h and 24 h post-infection. The DE genes in cluster J were significantly down-regulated by 12 h and remained at similar levels until 24 h post infection. Overall, genes in clusters A, B, C and D are up-regulated, while genes in clusters H, I and J are down-regulated at 24 h post infection. For each system, we identified DE genes that were involved in 10 and more pathways and those that were exclusively included in the systems. In the cellular processes and the organismal systems, we found that more than half of the multi-functional DE genes (17/30 = 57% for the former system and 40/70 = 57% for the latter system) had five point expression patterns clustered in H, I and J, while about half of the exclusively expressed DE genes (12/24 = 50% for the former system and 15/31 = 48% for the latter system) were grouped in clusters A, B and C. In contrast, half of the multi-functional DE genes (6/12 = 50%) in the genetic information processing were clustered in B, C and D, while 12 of 21 (57%) exclusively expressed DE genes in the same system fell into clusters H, I and J. Interestingly, the majority of both multi-functional DE genes (28/55 = 51%) and exclusively expressed DE genes (28/46 = 61%) in the metabolism systems had the same expression trends clustered in A, B and C. A total of 19 DE genes identified in PAMs infected with PRRSV are involved in at least 10 human diseases and most of them (17/19 = 89%) were clustered in H, I and J. System-specific expression patterns were not identified in the environmental information processing and human disease systems because a limited number of multi-functional and exclusively expressed DE genes were identified in these systems.

In addition to those system-based features in PAMs infected with PRRSV, we also observed other specific features related to PRRSV infection. Among 699 DE genes discovered in the present study, 206 were commonly down-regulated genes at different infection time points (Figure 4C) and they were involved in many function processes. Our data showed the signal transduction genes (*NFKB1*, *NFKB2*, *JUN*, *JUNB* and *FOS*) that trigger immune and inflammatory responses were significantly decreased. As a result, the proinflammatory cytokine genes (*IL-1A*, *IL-1B* and *TNF*) and chemokine genes (*CCL23* and *CCL3L1*) were down-regulated. The receptors of cytokines and chemokines (*IL1RN* and *CCRL1*) were also down-regulated. The combined effects may allow PRRSV to avoid an effective immune response. In addition, complement system activation seemed to be blocked during PRRSV infection. *C1QB*, *C3*, *C1QC,* and *FCN2*, activators of the lectin pathway of the complement system, were down-regulated [34]. The Heat shock proteins (HSPs) genes (*HSP90AA1*, *HSP90AB1*, *HSP90B1*, *HSPA1A*, *HSPA1B*, *HSPA5*, *HSPA6*, *HSPA8*, *HSPB1* and *HSPH1*) were negatively regulated during PRRSV infection. Heat stress proteins interact with viral proteins and enhance development of innate and adaptive immune responses against invading pathogens [35]; therefore, the down-regulation of HSPs in PRRSV-infected PAMs may have resulted in a weakened innate immune response. In addition, a recent study has identified that HSPA5 closely associates with PRRSV nsp2 and as such may be involved in PRRSV replication complexes [36].

The collection of MHC II genes (*SLA-DMB*, *SLA-DOA*, *SLA-DQA1*, *SLA-DQA2*, *SLA-DQB1*, *SLA-DRA*, and *SLA-DRB1*) and cell surface molecules (*CD74* and *CD83*) that help MHC II bind antigen were all down-regulated. PRRSV inhibits cell death or apoptosis, which allows a prolonged infection [37]. Negative regulation of apoptosis or cell death genes (*IER3*, *CFLAR*, *YWHAZ*, *PIM2*, *ANXA4*, *HMOX1*and *BAG3*) were also down-regulated. *CD163* is a PRRSV receptor that takes part in the internalization and uncoating of the virus. Down-regulation of CD163 could reduce PRRSV replication [38]. On the other hand, only 130 DE genes were commonly up-regulated at all four infected time stages. In particular, the up-regulated genes are involved in regulation of apoptosis and cell death (*MAEA, BNIP3, IDO1, GPX1, EI24,* and *TIAL1)* and oxygen metabolism (*ACE*, *VEGFA*, *BNIP3* and *EGLN2*) (Figure 4B). Superoxide dismutases (*SOD1* and *SOD2)* involved in the ubiquitin - proteasome pathway were significantly up-regulated, and the genes that encode proteasome subunits such as *PSMB1*, *PSMB2* and *PSME2* were also increased. Cyclooxygenase genes (*COX1* and *COX2*) which participate in virus replication and the regulation of inflammatory response following viruses infection [39] were up-regulated at four time points post-infection. The S100 family gene *S100A6* is involved in actin and tubulin cytoskeleton organization. Interferon regulatory transcription factor *IRF3,* which regulates IFN-β expression was activated at all four stages.

PRRSV affected expression of genes in PAMs at critical time points after infection. Interestingly, at 6 h post-infection, most of the affected genes were down-regulated, while many genes were up-regulated at 24 h post infection. At 6 h post infection, the energy metabolism related genes (*ATP5G2*, *COX17*, *COX5B*, *GSTP1*, *NDUFAB1* and *NDUFS2)* were suppressed. Transcriptional and protein synthesis genes (*H1FX* and *OBFC2B)* were also down-regulated. The antiviral gene *BST2*, which encodes the protein tetherin could directly restrict various viruses; however, *BST2* abundance was reduced by PRRSV infection [40], [41]. In addition, PRRSV infection of PAMs decreased the viral replication regulator ANAPC11 to control virus replication. Consistent with a previous report [42], genes in the DUSP family (*DUSP1*, *DUSP2*, *DUSP5* and *DUSP6*), which regulate MAPK signaling, were down-regulated at four time points post-infection. At 24 h post infection, when the viral replication is exponential, an increasing amount of protein is needed to assemble whole virions. Consistent with this protein need, PRRSV up-regulated expression of several ribosomal protein genes (*RPL15*, *RPL29*, *RPL34*, *RPL36A*, *RPL41*, *RPL9*, *RPLP0*, *RPLP1*, *RPS13*, *RPS16*, *RPS18*, *RPS19*, *RPS27L* and *RPS7*) to make translation more productive. Selected antiviral genes (*IFI6*, *IFIT3* and *IFITM3)* were also up-regulated, indicating that PAMs were attempting to control the viral infection. *S100A9,* another member of the S100 family that is involved in induction of the inflammatory response common to many pathogen infections, was also significantly up-regulated. This gene and family was also found to be in the top ten up-regulated genes in the tracheobronchial lymph nodes of pigs infected with highly pathogenic PRRSV rJXwn06 versus control at 14 days post infection [43]. Genes that were down-regulated genes at 24h post infection included inflammatory and anti-pathogen genes (*CSF1* and *USP2)* and the cell apoptosis and death genes (*MICB*, *BUB1B*, *ITGB2*, *UBB* and *BIRC3*).

The host-pathogen interaction is another feature to discuss as a virus develops many ways to manipulate host gene expression [44]. During PRRSV infection, PRRSV modulates the transcription and translation of the host cell to enable propagation and survival of the virus [27]. PRRSV infection of PAMS resulted in suppressed expression of IL1α, IL1β and TNF-α, which are important proinflammatory cytokines known to elicit innate immunity to restrict virus replication. Normally, virus infection may enhance *IL-1α*, *IL-1β* and *TNF-α* expression that consequently inhibits virus replication [45]. Lack of early *TNF* expression may be a method that PRRSV utilizes to evade aspects of the innate host immune response. Therefore, PRRSV evades the early lines of defense by effectively blocking the expression of important innate/inflammatory genes. The abundances of *IL1*, *IL6* and *TNF* were 10–100 times less in PRRSV single inoculated pigs than PRRSV-LPS inoculated pigs [46]. Similar results were observed in PAMs during PRRSV infection [47]. Our data showed that PRRSV infection restricts expression of these genes from 6 h to 24 h post infection. The complement pathway supports phagocytosis through opsonization and subsequent elimination of pathogens [48]. Down-regulated C3 and other complement pathway genes may weaken antiviral ability of phagocytic cells such as PAMs. Similar results were observed in PAMs during HP-PRRSV infection *in* *vivo* [49]. However, Xiao et al. showed that the complement system was activated in lung tissue during PRRSV infection, which may have caused severe lung damage [50], [51]. In the present study, the IFN-stimulated genes (*IFIT1*, *IFIT3*, *MX1*, *ISG15*, *OAS1*, and *IFI6*) were dramatically increased at 16 h and 24 h post infection, which should trigger powerful antiviral functions. This represents a positive signal for a host cell responding to PRRSV infection. Previous studies have shown that very low or negligible levels of IFN-α are produced upon PRRSV infection in pulmonary alveolar macrophages (PAMs) and PRRSV permissive monkey kidney cells (MARC-145) *in vitro* [52], [53]. IFN-α production in the lungs of pigs acutely infected with PRRSV was either almost undetectable or 100- to 200-fold lower than that induced by porcine respiratory coronavirus (PRCV) [54], [55]. PRRSV has also been found to suppress IFN-α production by transmissible gastroenteritis corona virus (TGEV), a known inducer of IFNs in infected alveolar macrophages [52]. At the same time, externally provided IFN-α or IFN-β have been able to reduce viral replication in cultured alveolar macrophages [52], [56]. PRRSV is thought to suppress type I IFN expression and block its signaling by interfering with STAT1/STAT2 nuclear translocation [57]. The virus was also found to inhibit the dsRNA-mediated up-regulation of IFN-β gene transcription [53]. A microarray analysis of PAMs infected with Lelystad virus (European type PRRSV) showed no significant change in the IFN-α from the control at 12 h post-infection [19]. IRF3, which plays an important role in activating type I interferon was up-regulated at all infected time stages. However, we did not detect differential expression of type I IFN over the course of infection. It is possible that type I IFN might have been induced before 6 h, which was our first PAM collection time. It has been shown the PRRSV can trigger the activation of IRF-3 as well as induce IFN production at 24 h post infection but the activities are much lower than those triggered by Poly(I:C) and PRRSV nsp1 antagonizes IFN production through the TLR3 and RIG-I pathways and down-regulates the protein level of IRF-3 [58]. Pre-treatment of PAMs with LPS down-regulated expression of CD163, a PRRSV receptor involved in PRRSV uncoating, and restricted PRRSV replication [38], [59]. In the present study, CD163 was significantly decreased after PRRSV infection, which indicates that viral evasion methods in PAMs were actively induced.

In conclusion, our current study revealed the largest known set of 699 DE genes in PAMs challenged with PRRSV, which are involved in six biological systems, 60 functional categories and 504 pathways. The major reactomes of PAMs responding to PRRSV infection included cell growth and death, transcription processes, signal transductions, energy metabolism, immune system and infectious diseases. In particular, PRRSV infection dramatically minimized pathway functions involving the actin filament based processes, anti-apoptosis, positive regulation of cell communication, protein folding, protein processing in endoplasmic reticulum, signaling by MAPK, NGF and GPCR, GPCR ligand binding, positive regulation of signal transduction, metabolism of lipids and lipoproteins, positive regulation of developmental processes, response to unfolded protein, developmental biology, axon guidance, positive regulation of immune responses, response to organic substances, response to hormone stimulus and response to abiotic stimulus in PAMs. However, PRRSV invasion maximized pathway functions related to membrane organization, lysosome, gene expression, nonsense-mediated decay, translation, translation elongation, translation initiation, ribosome, translation termination, SRP-dependent cotranslational protein targeting to membrane, transmembrane transport of small molecules, generation of precursor metabolites and energy, the citric acid cycle and respiratory electron transport, respiratory electron transport, ATP synthesis by chemiosmotic coupling, and heat production by uncoupling proteins, oxidation reduction and oxidative phosphorylation, metabolism of proteins, hexose metabolic processes, glucose metabolic processes and vesicle docking during exocytosis in PAMs. Overall, PRRSV took control of PAMs in the course of a 24 hour infection, but the host started to fight back using its autophagy mechanisms.

## Materials and Methods

### Ethics Statement

The animal use protocol was reviewed and approved by the Institutional Animal Care and Use Committee (IACUC) of the National Animal Disease Center-USDA-Agricultural Research Service.

### Cells, Virus Infection and SAGE Analysis

The experiments were conducted as previously described [60], [61]. In brief, PAM cells were harvested from three clinically healthy, PRRS-negative gilts 6–8 weeks of age and tested free by PCR for both porcine circovirus and *Mycoplasma* spp. Primary PAM were isolated, cultured, and infected, as previously described [62]. Aliquots of PAMs were then frozen and stored in liquid nitrogen separately for all three pigs. After establishing PAMs in culture, cells were infected with PRRSV strain VR-2332. To achieve a near synchronous infection, flasks containing adherent PAMs were infected at a multiplicity of infection (MOI) of 10 in chilled media and incubated at 4°C for 1 hour to allow for virus binding, but not entry into the cell. Pre-warmed media was added and the cells placed at 37°C, 5% CO_2_ until collected for RNA isolation. Total cellular RNA was prepared using the Qiagen RNeasy mini kit (Qiagen, Valencia, CA) according to the manufacturer’s instructions. Cell samples were collected from each PRRSV-infected PAM flask at 0, 6, 12, 16 or 24 hours after infection. Total cellular RNA from mock-infected PAMs was collected at 0 and 24 hours. Equimolar amounts of total RNA from the PAMs of each pig at each time point were then pooled to make SAGE (serial analysis of gene expression) libraries using *Nla*III as the anchoring enzyme and *BsmF*I as the tagging enzyme [63]. The SAGE libraries provided the population means of the transcript abundance levels for each time point. SAGE clones were amplified and sequenced using a high-throughput sequencing pipeline with an ABI 3730 automated sequencer and ABI chemistry (Applied Biosystems Inc., Foster City, CA). The SAGE libraries with tag counts were submitted to GenBank GEO and have the accession number GSE10346.

### Detection of Differentially Expressed SAGE Tags

We assumed that the counts of the i^th^ SAGE tags, 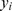, followed a Poisson distribution defined as:
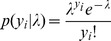
(1)


We then let 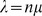 where 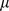 was interpreted as the “true” frequency that the i^th^ SAGE tag being expressed, among *n* expressed SAGE tags in total, such that
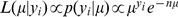
(2)


Here, both 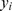 and 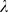 are not comparable between statuses, because the library sizes can vary. We can however, compare 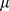
’s among statuses (i.e., libraries) because they quantify the underlying “true” frequencies of SAGE expression.

Within the Bayesian framework, we assumed a conjugate Gamma prior distribution, Gamma (a, b), for the parameter 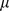, then, the posterior distribution of 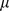 is also a Gamma density with parameters 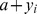 and 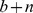,
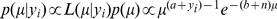
(3)


Now, we considered 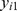 and 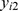 are counts of the *i* ^th^ SAGE tag at two time points, 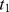 and 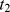, respectively, and 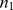 and 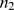 are the total numbers of SAGE tags measured at these two time points. Under the assumption of heterogeneity 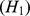, the means at the two time points are different, that is, 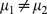. Then, the likelihood function is
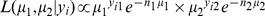


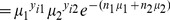
(4)


Given a Gamma prior distribution, Gamma (a,b), to 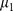 and 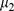, we showed that their posterior distributions are also Gamma:
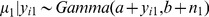
(5a)

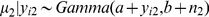
(5b)


Under the assumption of homogeneity 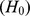, 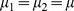, the likelihood function was
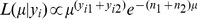
(6)


Then the posterior distribution of 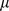 was:
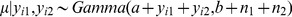
(7)


In Bayesian framework, this hypothesis test that contrasts both models (hypotheses) was conducted using the Bayes Factor [64].

Differential gene expression is commonly measured by computing log ratios. In the present study, we similarly computed log ratios (pLR) of posterior frequencies of SAGE tags between each of the treatment time periods (6 h, 12 h, 16 h, or 24 h) and the normal status, as follows:
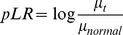
(8)


Alternatively, differential gene expression between two time points can be evaluated by computing the following probability based on posterior samples:
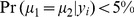
(9)


Hence, we numerically constructed the 95% highest posterior density intervals (95% HPD) for 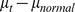, and differential expression of a SAGE tag was claimed to be true if one of the following criteria held, or false otherwise:



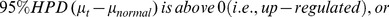
(10a)




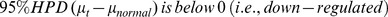
(10b)


In the present study, we employed both criteria, (8) and (10), to identify differentially expressed (DE) SAGE tags.

### Assignment of DE Tags to Genes

We downloaded a total of 52,121 porcine mRNA sequences from the GenBank database at National Center for Biotechnology Information (NCBI). A Java program was developed to identify the 3′ most *Nla*III cut site for each mRNA sequence and collect the sequence of 10 nucleotides following the anchoring enzyme cut site. The process resulted in 48,988 tags collected from 52,121 mRNA sequences. Interestingly, 95 mRNA sequences had the same AAAAAAAAAA tag and were subsequently deleted from the analysis. We also simply assumed that among the remaining tags, any repeated mRNA sequences with different accession numbers belonged to the same gene. By excluding all repeats, we compiled a list of 26,745 unique tags for different pig mRNAs. The unique mRNA tags were then merged with the DE SAGE tags identified above to determine DE mRNAs of PAMs infected with PRRSV. The mRNA sequences were then annotated for orthologs in the human genome against the Refseq database, as the human genome has been well annotated.

### Identification of DE Genes for Pathway Analysis

In order to select DE genes for pathway analysis, we arbitrarily required that each DE gene had at least one time point (6 h, 12 h, 16 h, and 24 h, respectively) that was at least a 2 fold change and 100 tags per million (TPM) different from the 0 h mock-infected control. In addition, we also required that each DE gene was at least 1.5 fold different between the 24 h-infected and the 0 hour mock-infected cells. Generally speaking, we assumed that a cell should express ∼10,000 genes at a given time [65]. As such, each gene should average 100 TPM when a million SAGE tags are sequenced. Therefore, we considered 100 TPM as a minimum requirement to signify a change of functional importance for a DE gene. The associated pathways of all DE genes were identified using DAVID, (http://david.abcc.ncifcrf.gov/home.jsp), KEGG Pathway (http://www.genome.jp/kegg/pathway.html), and Reactome (http://www.reactome.org/ReactomeGWT/entrypoint.html) databases. The DE gene clustering was performed using the self-organizing map (SOM) as described previously [65]. A SOM is a type of artificial neural network that uses unsupervised learning to produce a low-dimensional, discretized representation of the input space of the training samples, called a map. It consists of components called nodes or neurons. Each node is associated with a weight vector of the same dimension (as the input data vectors and a position in the map space). To place a vector from data space onto the map, the method first finds the node with the closest weight vector to the vector taken from data space. Once the closest node is located, it is given the values from the vector taken from the data space.

SOMs operate in two modes: training and mapping. Training builds the map using input examples (data), which features competitive learning, and mapping automatically classifies a new input vector. When a training example is fed to the network, the method computes its Euclidean distance to all weight vectors. The neuron with weight vector that is most similar to this input is called the best matching unit (BMU). The weights of the BMU and neurons close to it in the SOM are adjusted towards the input vector. The magnitude of the change decreases with time and with distance from the BMU. The update formula for a neuron with weight vector *W* ***_v_***(*t*) is as follows:
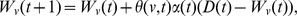
where α(t) is a monotonically decreasing learning coefficient and ***D***(*t*) is the input vector. The neighborhood function ***θ***(*v*, *t*) depends on the distance between the BMU and neuron *v*. The neighborhood function shrinks with time. At the beginning when the neighborhood is broad, the self-organizing takes place on the global scale. When the neighborhood has shrunk to just a couple of neurons the weights are converging to local estimates. This process is repeated for each input vector for a number of cycles (denoted as *λ*, usually in a few hundreds of cycles). Typically, SOMs with a small number of nodes behave similarly to K-means, whereas larger SOMs can rearrange data in a way that is fundamentally topological in character [66].

## Supporting Information

Table S1 PRRSV tags, row counts and TPM detected.
(XLSX)

Table S2 DE tags, row counts and TPM detected post Bayesian analysis.
(XLSX)

Table S3 DE genes assigned by SOM analysis to clusters based on their expression trends regardless of their fold-change magnitudes along time points (0 h, 6 h, 12 h, 16 h and 24 h).
(XLSX)
